# Towards dealing with commonly occurring requirements engineering process issues during software development outsourcing

**DOI:** 10.1371/journal.pone.0269607

**Published:** 2022-07-14

**Authors:** Javed Iqbal, Rodina B. Ahmad, Fazal-e- Amin, Muhammad Shoaib, Mohd Hairul Nizam Nasir

**Affiliations:** 1 Department of Computer Science, COMSATS University Islamabad, Islamabad, Pakistan; 2 Department of Software Engineering, Faculty of Computer Science and Information Technology, University of Malaya, Kuala Lumpur, Malaysia; 3 Department of Software Engineering, College of Computer and Information Sciences, King Saud University, Riyadh, Saudi Arabia; 4 Department of Information Systems, College of Computer and Information Sciences, King Saud University, Riyadh, Saudi Arabia; National Textile University, PAKISTAN

## Abstract

Due to specific advantages, the volume of Software Development Outsourcing (SDO) is rapidly increasing. Because of challenges arising from the Requirements Engineering (RE) process, the anticipated benefits of SDO are not achieved in case of several projects. The objective of this research work is to recommend RE practices for addressing the commonly arising RE process issues in the case of SDO. For this reason, a thorough literature review has been undertaken, as well as two questionnaire surveys have been performed with skilled SDO industry practitioners. The surveys have been done by utilizing semi-supervised style and employing Convenience Sampling method. The 50 percent rule and a four-point Likert Scale have also been used to determine the advantages of RE practices for dealing with the issues. A comprehensive list of 147 RE practices has been extracted by conducting a Focus Group session. Furthermore, the 147 RE practices have been ranked by applying Numerical Assignment and Hundred Dollar Techniques during two Focus Group sessions. The detection and adaptation of RE practices aids in enhancing the SDO RE process, evading SDO failures, and achieving the associated SDO advantages.

## 1. Introduction

Information Technology Outsourcing (ITO) is the subcontracting of particular services or the use of external resources to undertake IT functions [1]. The volume of ITO is steadily growing. The IT services outsourcing business was of worth $520.74 billion in 2019 and is expected to grow to $937.67 billion by 2027 [2].

Software Development Outsourcing (SDO) is a sort of ITO in which a customer contracts out some or all the software development operations to the vendor (s) [3–6]. For both advanced and developing countries, SDO generates a win-win state [7]. Software development projects are being outsourced to India, Russia, and China by European companies [8]. SDO options include onshoring or domestic outsourcing, nearshoring, offshoring, Distributed Software Development (DSD), and Global Software Development (GSD).

SDO offers benefits such as reduced cost, better capabilities, access to cutting-edge technologies, shorter completion times, process enhancement, novelty, risk mitigation, optimum use of internal resources, and institutional deficiencies such as inept management, inexpert staff, and resource scarcity [9–11]. However, a significant portion of SDO initiatives fails to deliver the expected advantages [8, 12–14]. Failures causes are frequently pin down to the Requirements Engineering (RE) process for SDO [8, 15–17]. This is not unexpected, given that RE is the most important stage of the software development life cycle and has a considerable impact on the other software development activities [18, 19]. This is due to the fact that flaws left untreated during the RE phase frequently cascade into subsequent phases. As per findings of a study, insufficient requirements specification causes planning and control challenges [17]. According to previous research, fixing a RE problem later in the software development life cycle can cost up to 100 times more than fixing a coding error [20].

The requirements elicitation, analysis, and negotiating stage, as well as the description, modelling, validation, and management phases, are all challenging and complicated [21–23]. Because the players in the SDO are geographically dispersed, the RE process issues are multiplied [15, 21, 24]. The geographical diffusion of stakeholders has an impact on the RE process’s numerous activities, causing communication, knowledge management, cultural, and coordination issues. Consequently, to attain the projected advantages of the SDO, the issues of the RE process for SDO must be tackled. To tackle the issues, the pertinent RE practices must be discovered for enriching the SDO RE process as a well-specified RE process is vital to the fruitful outsourced software development projects with regard to price, timing, and quality [18]. Considering this, the purpose of this research work is to respond to the following Research Questions (RQs).

RQ1: Which are the RE practices, grounded on existing literature, for dealing with the issues of the RE process in the context of SDO?

Sommerville and Sawyer have provided an adequate number of RE practices to overcome the problems with the traditional RE approach [22]. To determine empirically which of these RE practices are crucial to tackle the RE process issues for SDO, the second RQ is:

RQ2: Which of Sommerville and Sawyer’s proposed RE practices are important for addressing issues with the RE process for SDO?

For a thorough and successful research, it is necessary to include the viewpoint of the industry. Hence, the third RQ is:

RQ3: Which RE practices do SDO professionals use to address the issues with the RE process for SDO?

To know the importance of RE practices for addressing SDO RE process issues, the practices need to be ranked. Therefore, fourth RQ is:

RQ4: Which are ranks of the RE practices with respect to importance of the practices for addressing issues of SDO RE process?

The remainder of the article is structured as follows: Section 2 covers relevant work, Section 3 describes research methodology, Section 4 presents results and discussion, Section 5 provides ranking of RE practices and Section 6 wraps up the research work.

## 2. Related work

The RE process, for scenarios in which stakeholders are scattered across multiple sites, has been studied from many perspectives in the current literature. One of the perspectives focuses on the issues that arise throughout the RE process.

The geographical dispersal of stakeholders has a substantial influence on RE [25]. In such cases, the teams have to confront obstacles during requirement elicitation and negotiation. In the case of GSD, certain issues have an impact on the efficacy of requirements elicitation techniques: i) inadequate scope management, ii) poor comprehension of requirements, and iii) requirements’ volatility. An RE elicitation strategy, in the GSD context, has been developed for addressing such issues [26].

The implications of inadequate RE on outsourced software projects have been explored from the developers’ viewpoint [7]. To achieve this objective, a questionnaire survey was done with 57 developers who have dealt with SDO and have worked for 8 small and medium software companies. Developers must cope with too many requirements’ modifications, reduced designs, extended development stages, and an unanticipated number of deliverables, according to data analysis. In [27], some findings concerning the distributed RE process in the context of GSD have been derived. The dispersed environment has certain distinct characteristics, such as distance between stakeholders, time zone variations, and cultural diversity, all of which have an impact on the RE process. The impact of these issues on the distributed RE process is briefly described in this paper, and it is suggested that a distinct RE process must be developed to accommodate the dispersed environment.

Inadequate communication, time zone disparities, diverse cultures, and poor knowledge management all hinder the elicitation of requirements in the context of GSD [28]. A framework named RE-GSD (Requirement Elicitation for Global Software Development) has been suggested to overcome such issues.

An industrial perspective report [29] highlights the issues that impact the management of changing requirements in the outsourcing scenario. Finally, the study presents a conceptual framework for RE Systems. Another article about GSD [30] focuses on Requirements Change Management (RCM) and provides a framework to overcome RCM challenges, primarily communication challenges. One more experimental study [16] analyses and tackles requirements management concerns in the case of globally distributed software development projects.

The focal point of another research is Requirements Understanding in GSD [31]. Authors aim to discover: i) Various elements which generate problems for Requirements Understanding in the GSD scenario, and ii) Resolutions to cope with those problems. Finding demonstrates that the usage of the NetMeeting web-based technology, which was used to enable requirements communication between two remote sites, improves the dispersed RE process [32].

Multinational initiatives with a huge number of collaborators, user, and developer groups; are differentiated by a wide range of backgrounds and abilities [33]. Data collection and validation during the RE in this diversified context need efficient processing strategies to deal with large amounts of data and for controlling inconsistencies during online data gathering. PBURC (Pattern-Based Unsupervised Requirements Clustering) is a framework that has been developed for this purpose. Another research [34] proposed the usage of MAS (Multi Agent System) architecture to reduce the challenges that occur throughout the distributed RE process, particularly within verification and validation tasks. Exploratory research was done to better understand the complexity inherent during the RE process in the context of GSD projects by examining the case of 24 virtual teams that participated in five-week activities of identifying requirements [18]. When partners are geographically spread, a paradigm called the V model has been developed for generating and choosing requirements for a software release [35].

In another study [36], knowledge transfer and reuse in the global RE perspective are also examined. Distance between partners and cultural differences have an impact on knowledge exchange. Improper reuse in RE is a result of mistrust and protectionism. The findings of an exploratory investigation on the role and relevance of the human moderator during dispersed RE, have also been presented [37].

The 43 most common RE process issues in the SDO scenario have been identified, classified, and prioritized based on their frequency of recurrence [38]. The 25 elements that can influence the offshore SDO RE process have also been discovered and verified [39]. With efficiency and cost reduction in mind, Ahmed and Abdulrahman have investigated the impact of localized decisions, about requirements, on software customization in the DSD scenario [40]. The RCM process for dispersed agile software development has been streamlined using a methodology and a tool [41]. Another study [42] presents 15 issues for quality requirements the in the case of huge DSD agile projects, as well as 13 processes and 9 strategies for dealing with the challenges. In the context of GSD, a well-ordered domain ontology has been recommended to aid the RCM process [43]. For the GSD scenario, the 30 RCM problems have been explored and classified [44]. The 23 elements that lead to an effective RCM for GSD, have been uncovered [45]. In another research work [46], a three-stage technique has been presented to aid the RCM process in the GSD scenario. The elements that facilitate the implementation of appropriate requirements in the GSD scenario, have been discovered and examined [47]. Two frameworks have been suggested and validated to study the significance of project management exclusively for RE and then RCM procedures in the context of GSD [48]. The 218 risks linked with the RE process in GSD scenario, as well as 146 measures to mitigate those risks, have been outlined [49]. A paradigm has been proposed to investigate the impact of geographical, intercultural, and spatial disparities on communication during RCM process in the GSD scenario [50]. Thirteen strategies have been discovered to support efficient communication for requirements elicitation in the context of GSD projects [51]. In GSD scenario, RCM obstacles have been discovered, confirmed, and quantified [52]. A methodology has been designed to evaluate GSD companies’ preparedness for the RCM process in GSD scenario [53].

A two-phase approach for prioritising requirements in the GSD environment, has been presented depending on three criteria: weight, vote, and priority of partners [54]. A model has been created and tested to investigate the impact of fluctuating requirements on the sustainability of GSD initiatives [55]. Based on company scale and location, the success elements that are necessary for successful communication across the requirements elicitation in the GSD scenario have been examined [56]. A mechanism for specifying and validating requirements has been established by generating a requirements graph, particularly in GSD scenario [57]. A block-chain dependent approach has been presented to deal with the requirements discrepancies in GSD context [58]. With regard to business function, scale, and experts’ ability, the 20 obstacles in the context of GSD RE scenario have been identified and confirmed [59]. The 15 problems for RCM in the offshore SDO scenario have been discovered [60]. The 14 problems for reusing requirements in the case of huge agile DSD projects, as well as 10 approaches to solve those problems have been recommended [61]. Focusing on 32 communication problems and 28 RE practices, a paradigm has been presented to tackle communication-related problems for the RE process in GSD scenario [62].

Thus, an examination of recent research reveals the fact that studies just partly resolve the SDO RE issues or partly present the RE practices to tackle SDO RE issues. To our knowledge, no research has ever presented a complete list of RE practices for dealing with the SDO RE process issues. As a result, the focus of this study is to establish an exhaustive list of RE practices to tackle the most prevalent SDO RE process issues that lead to SDO failures.

## 3. Research methodology

The committee for candidature defence has authorized this research work, which is part of my PhD study. This research’s only human-associated subject matter is questionnaire surveys. The verbal agreement of prospective respondents or their corresponding organizations was requested prior to performing the surveys. In this study, no private data has been disclosed or examined in any way. The replies have been stated in a collective way. Individuals’ and organizations’ anonymousness and secrecy have been absolutely secured in this way.

**Fig 1:**
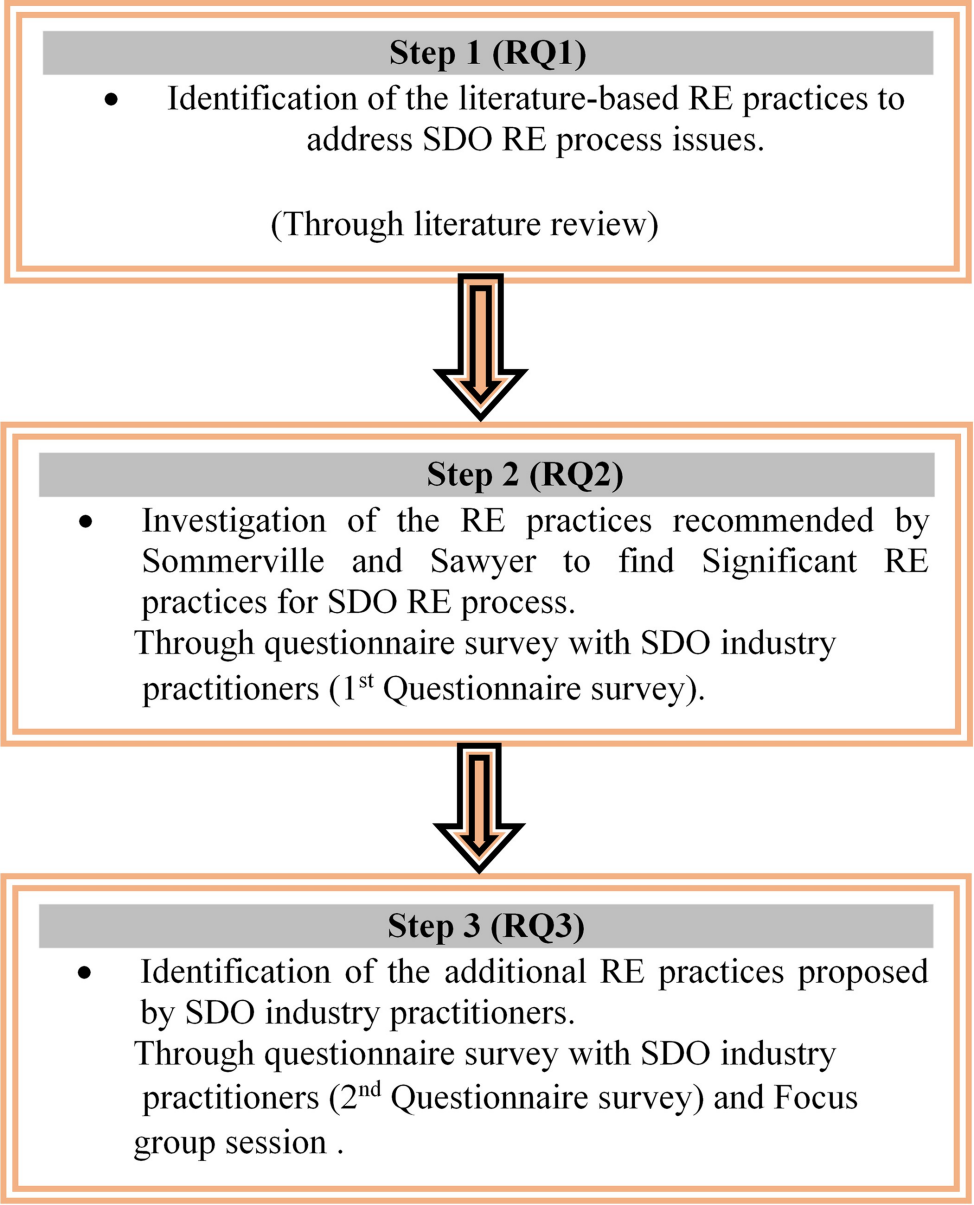
Steps for identifying RE practices to tackle SDO RE process issues.

To achieve research objectives, employed research methods are: Systematic Literature Review (SLR), questionnaire survey, 50% rule and focus group session. Fig 1 depicts research methodology.

Thus, three steps have been performed to meet this research works’ objectives. The sections 3.1, 3.2, 3.3 and 3.4 present introduction to employed methods.

### 3.1. Systematic literature review

The objective of a SLR is to find, examine, and synthesize all available research on a particular research question, subject, or area of focus [63]. Barbara Kitchenham’s technique [64] has been used to conduct the SLR in this study like the previous studies [65, 66].

### 3.2. Questionnaire surveys

Both questionnaire surveys used in this study have been done utilising a semi-supervised technique, in which the survey’s purpose, questionnaire structure, and numerous questions about the questionnaire are made explicit to respondents prior to survey [67]. Semi-supervised techniques have been employed in this study, either via head-on encounters with participants or via use of the Computer-Assisted Telephone Interviewing (CATI) methodology [68].

During the first survey, some questionnaires were provided and filled out at face-to-face meetings, while others were sent through emails. The Drop-Off/Pick-Up approach [69] was used for the second questionnaire survey.

### 3.3. The 50% rule

The 50 percent rule states that a viewpoint is considered if at least 50 percent of those polled agree with it. The analogous guideline has been applied in other investigations as well [70–72].

### 3.4. Focus group session

Focus groups are used to collect information about a certain topic chosen by the investigator. Focus groups are meticulously designed debates that allow participants to obtain personal insight into a certain study topic [73]. Focus groups have been used in numerous investigations [74, 75].

### 3.5. First questionnaire survey

The survey research approach has been used to gather information on significant RE practices for SDO. The first questionnaire survey of the research work is based on Sommerville and Sawyer’s 49 RE practices [22] for six major areas of the RE process: elicitation of requirements, evaluating and negotiating requirements, requirements description, modelling requirements, validating requirements, and maintaining requirements. The survey research approach is seen to be a good way to collect both qualitative and quantitative data [76]. Like Wohlin and Aurum [77], this survey was designed and conducted using the guidelines described in research [78].

A cross-sectional investigation was conducted for this survey. SDO practitioners received 130 surveys in total. Seventy (70) questionnaires were delivered and filled out at face-to-face meetings, while Sixty (60) questions were distributed and filled out over emails. The survey was carried out using either a semi-supervised approach [67], in which respondents were directed during face-to-face sessions, or the CATI technique [79]. In research, the CATI approach is commonly used [80–82].

The term "significant" refers to something that is "important enough to make an impact" [83, 84], therefore, significant RE practices means the RE practices which effect RE process.

### 3.6. Second questionnaire survey

The survey was designed and conducted using the guidelines described in research [78]. A cross-sectional investigation was conducted for this survey. The Drop-off/Pick-up approach was used to distribute 200 questionnaires. Various studies employed Drop-off/Pick-up approach [85, 86]. The survey was carried out using a semi-supervised method [67]. The survey’s goals, professionals’ assumptions, questionnaire style, and respondents’ questions were all clarified using the CATI approach [79].

## 4. Results and discussion

**Fig 2:**
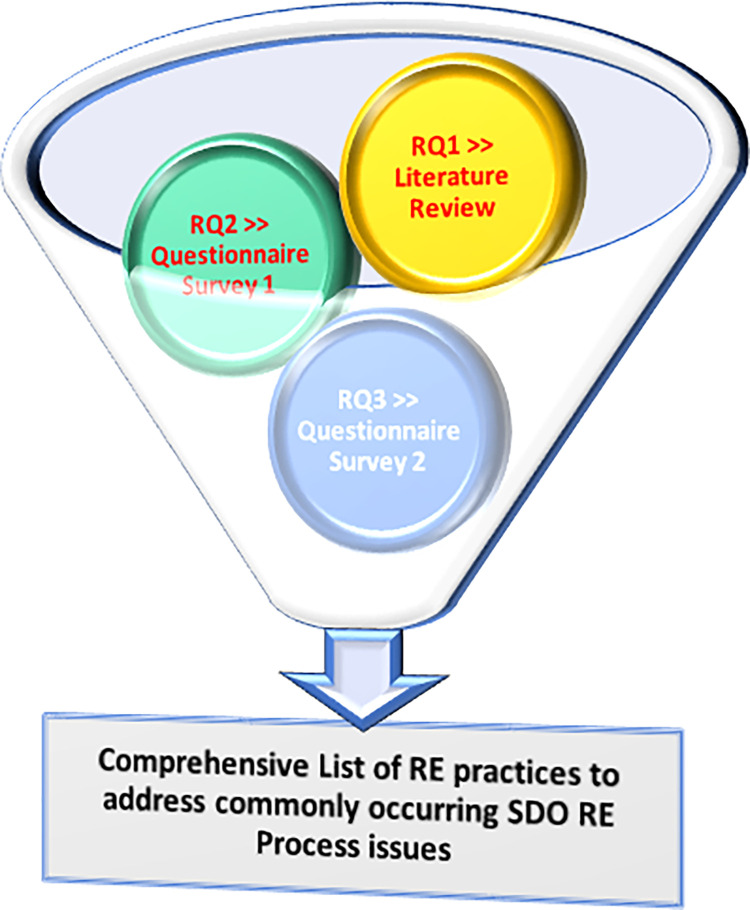
Data sources for detecting exhaustive list of RE practices.

This section summarises the findings of the research analyses by focusing on the three phases outlined in the section of research methodology. The procedure is depicted in Fig 2.

### 4.1. Step 1: Identifying RE practices, grounded on existing literature, to tackle the issues of RE process for SDO

SLR has been performed in the initial phase of this study.

#### 4.1.1. Systematic literature review

**Fig 3:**
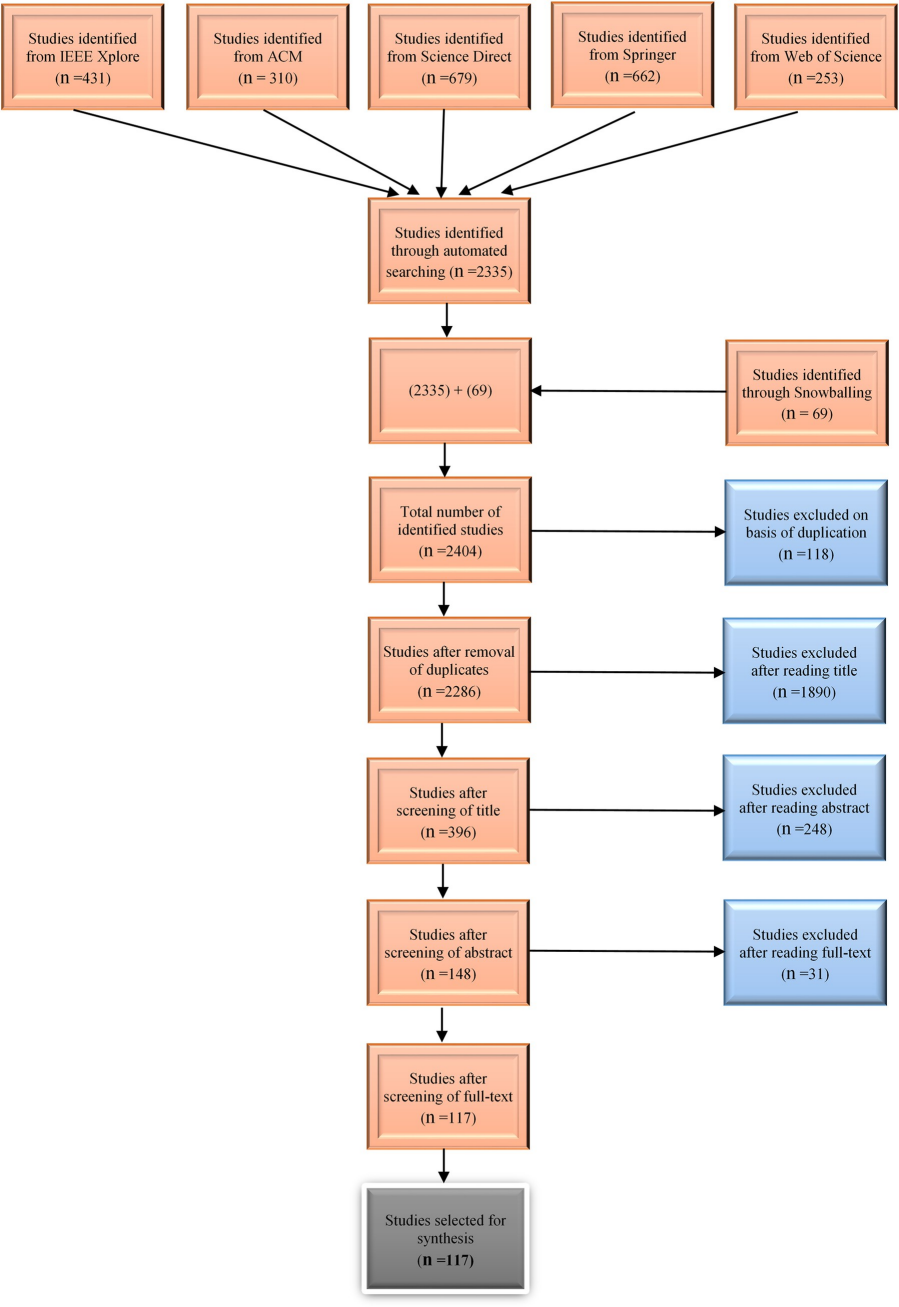
Studies selection procedure.

The targeted sources to find relevant studies are IEEE Xplore, ACM, Science Direct, Springer Link & Web of Science. Fig 3 depicts the SLR process. To eliminate bias, two other investigators have been engaged throughout the process, and each phase has been concluded after reaching an agreement.

After a rigorous review and screening procedure, 117 studies were chosen for extracting data.

#### 4.1.2. The RE practices to tackle SDO RE process issues

The 90 RE practices have been discovered, after a thorough examination of 117 studies, that can be applied to tackle the RE process issues in SDO scenario. The 90 RE practices denoted by the initials REPR_1_, REPR_2_, REPR_3_,…, REPR_90_ are as follows:

**REPR**_**1**_ = Putting in place the necessary infrastructure to support communication and guaranteeing that it is operational [87];

**REPR**_**2**_ = Promoting synchronous communication using chat rooms, phone conversations, and videoconferencing [87];

**REPR**_**3**_ = Adjusting to and comprehending various stakeholders’ cultures [87], means being familiar with a culture’s customs, values, ethos, and local language [5];

**REPR**_**4**_ = Choosing and utilizing a standardized communication language [88];

**REPR**_**5**_ = Concentrating on communication language improvement, such as English language classes [87, 89, 90];

**REPR**_**6**_ = Employing cultural liaisons [87, 89, 91] or intermediaries (people who are conversant with customer and vendor cultures) [92];

**REPR**_**7**_ = Creating a ’proximity training center’ in a time zone that is the same or somewhat different from the client’s zone [93];

**REPR**_**8**_ = Attempting to identify natural overlaps in work time [94];

**REPR**_**9**_ = Evaluating a person’s capacity to work ’around the clock’ [94];

**REPR**_**10**_ = Time-shifting (adjusting one’s working time to coincide with the work time of others) to attain time zone closeness, a variety of approaches for this purpose, are:

- Flextime (functioning on a flexible schedule to allow for overlapping).
- Overtime (functioning for additional time for overlapping).
- Telework (functioning for elastic schedules from home for overlapping).
- Long working days (allowing for work time overlapping at the beginning or finish of the day).
- Unrestricted working hours (employees decide their own operating time for overlapping and there are no standard work hours) [95];

**REPR**_**11**_ = Providing electronic message "drop in", remote phoning, and artefact distribution capabilities to distant practitioners’ rooms [96];

**REPR**_**12**_ = Enabling professional integration from the outset of the project, such as by holding face-to-face kick-off meetings to create personal interactions [21, 97];

**REPR**_**13**_ = Organizing regular visits to isolated locations in order to foster trust [18, 98, 99];

**REPR**_**14**_ = Encouraging direct contact amongst stakeholders [100];

**REPR**_**15**_ = Assuring that stakeholders are introduced to each other from the start of the project [101];

**REPR**_**16**_ = Facilitating interaction in the client’s original tongue [5];

**REPR**_**17**_ = Advising on the usage of groupware tools [99];

**REPR**_**18**_ = Attempting to convince stakeholders that disclosing concerns or sharing information would have beneficial repercussions rather than adverse effects [94];

**REPR**_**19**_ = Organizing video or teleconferences on day-to-day, weekly, semi-monthly, or monthly basis such that no or a few awkward hours exist for all the partners [98];

**REPR**_**20**_ = Organizing requirements engineering sessions by:

- Using a human moderator and a rich communication medium that allows data, audios, and videos to be integrated.
- Making and sticking to an agenda.
- Identifying appropriate participants and notifying them on time to participate in requirements engineering sessions.
- Exchanging of reference materials in a timely manner to allow participants ample time to view the essential content.
- Giving attendees of requirements sessions access to resources (like emails, relevant papers, work artefacts, and so on) containing information on the requirements [21];

**REPR**_**21**_ = Setting up assertive governance at the project manager and team leader levels [102];

**REPR**_**22**_ = Keeping track of the directives in a certain order [102];

**REPR**_**23**_ = Personal and group obligations must be clearly stated and agreed upon [102];

**REPR**_**24**_ = Getting obviously defined and grasped requirements engineering processes [102];

**REPR**_**25**_ = Utilizing email as a communication verification tool because it preserves a written copy of contact [21, 89, 96];

**REPR**_**26**_ = Getting agreements in writing and adequately documented [103];

**REPR**_**27**_ = Creating an organizational structure with clear communication roles [91];

**REPR**_**28**_ = At the various levels of team, project, and management; creating peer-to-peer connectivity across remote locations [91];

**REPR**_**29**_ = Partly orchestrating inter-organizational procedures [91];

**REPR**_**30**_ = Providing open channels of communication amongst stakeholders with clearly defined responsibilities [91];

**REPR**_**31**_ = Assessing and communicating development on jointly approved artefacts on a regular basis [91];

**REPR**_**32**_ = Making use of an awareness support system to manage requirements, all partners ought to be able to obtain the following details:

- Specifications, justifications, and priorities of various requirements.
- Requirements dependencies, as well as design, code, and testing dependencies.
- Duties of every member of the team in relation to specific requirement(s) and contact particulars like email address and phone number.
- The people who took initiatives for the requirements.
- Issues relating to requirements, issues’ activators, state of issues’ resolution, and judgments made because of issues.
- Dates, times, and venues of the meetings, as well as the present stakeholders, debated issues and judgments done.
- Demands for change, change demand activators, state of every change demand, personnel engaged in making judgments, and judgments made [104];

**REPR**_**33**_ = Retaining senior professionals on the team and persuading those professionals to bridge the knowledge gap [105];

**REPR**_**34**_ = Putting in place a centralized communication system [105];

**REPR**_**35**_ = After each meeting, making a report of the proceedings. Any member of the team or moderator ought to outline which issues were presented at the meeting, what decisions were taken about every issue, still open issues, who is responsible for gathering more data, and whose guidance should be solicited in the event of each issue [106];

**REPR**_**36**_ = To use a Requirements Management System (to regulate and follow changes) that has the succeeding characteristics:

- Searching a list of requirements, extracting individual requirements, and organizing requirements according to certain criteria.
- Administration of the requirements modification process, assistance for requirements traceability, and development of various forms of requirements reporting.
- Acceptance of external documents via an interface.
- Managing several versions of requirements.
- Aid for carrying out various forms of analyses (e.g., impact analysis, knowing whether a requirement is orphan, status tracking).
- Limiting access and editing privileges to the list of requirements [29];

**REPR**_**37**_ = Notifying the pertinent partner regarding the change in requirements by:

- Communication technologies such as telephone, emails, and the internet.
- Using the system to send out automated notifications [107];

**REPR**_**38**_ = In the event that there are a large number of partners:

- Designating a person (communication channel) out of each organizational unit or group of requirements sources to collect requirements from that unit or group. The requirements are then transferred to an expert via communication channels, where they might be combined [108].
- Obtaining unanimity on requirements employing group elicitation approaches like Brainstorming, JAD, Focus groups, and requirements Workshops [25].
- Generating a consolidated requirements document that includes all of the requirements [108];

**REPR**_**39**_ = Adopting the following steps to address cultural challenges:

- (REPR_6_) Employing cultural liaisons [87, 89, 91] or intermediaries (people who are conversant with customer and vendor cultures) [92].
- Advising members of the team to tour other partners’ sites [109].
- Organizing cultural training sessions [109].
- Offering cross-cultural orientation sessions [109].
- Considering stakeholders’ cultural beliefs when determining female responsibilities [109].
- Introducing a ’Negotiated Culture,’ a negotiated culture created to respect all partners’ cultural standards [103].
- Identifying persons with expertise and familiarity with the customer’s culture to support for requirements discussion and definition [89].
- (REPR_4_) Choosing and utilizing a standardized communication language [88].
- (REPR_5_) Concentrating on communication language improvement, such as English language classes [87, 89, 90].
- The project manager or/and experienced team members planning and supervision of all the efforts that are carried out to cope with cultural differences [109];

**REPR**_**40**_ = Presenting the Equality Model (EM) for all partners, in which all partners are treated equally and can discuss each other’s preferences, religion, and social traditions. They can also exchange knowledge and make recommendations based on others’ perspectives and roles [110];

**REPR**_**41**_ = Defining the procedures, techniques, and policies that must be followed [111];

**REPR**_**42**_ = Knowledge exchange [111];

**REPR**_**43**_ = Observance of common hopes [111];

**REPR**_**44**_ = Possessing technological, managerial, and personnel capabilities for satisfying quality standards & meeting deadlines [5];

**REPR**_**45**_ = To inspire non-fluent or less confident partners to participate in the discourse, begin with an informal dialogue [112];

**REPR**_**46**_ = Making use of translation facilities:

- Utilizing human interpreter [112, 113].
- Utilizing real-time machine conversion facilities [113];

**REPR**_**47**_ = Utilizing scales to calculate the mean time it takes for hopes to be met. For example, providing a feature that determines the average time it takes a person or team to react to an email. If the mean time to respond is three days, the sender may anticipate receiving a response within three days [114];

**REPR**_**48**_ = Developing and utilizing a vocabulary and notations for requirements description [88];

**REPR**_**49**_ = Vendor managers can take the following steps to establish collaboration:

- Establishing team members’ roles and tasks, as well as constructing Organizational Charts that show their roles and obligations [115].
- Obtaining and administering the appropriate human resources using Resource Calendar [115].
- Appropriate task distribution [115].
- (REPR_28_) At the various levels of team, project, and management; creating peer-to-peer connectivity across remote locations [91].
- (REPR_29_) Partly orchestrating inter-organizational procedures [91].
- (REPR_30_) Providing open channels of communication amongst stakeholders with clearly defined responsibilities [91].
- (REPR_31_) Assessing and communicating development on jointly approved artefacts on a regular basis [91];

**REPR**_**50**_ = Achieving partners’ agreement on meeting attendance terms and conditions, as well as fulfilling timelines and obligations [116];

**REPR**_**51**_ = Identifying each team member’s function and suggesting who might interact with whom [16, 117];

**REPR**_**52**_ = In terms of decisions, keep in constant contact with customers by organizing:

- Meetings in person.
- Video conferencing [16];

**REPR**_**53**_ = Selecting a teammate that works beyond the typical business hours and responds to questions [118];

**REPR**_**54**_ = Educating on in what manner to:

- Make use of the available tools.
- Interact efficiently in a situation where partners are scattered at remote sites [18];

**REPR**_**55**_ = Giving prospective team members training about how to use relevant procedures, as well as associated tools and technology [119];

**REPR**_**56**_ = Performing six general steps for RE, in absence of any standard RE process [20, 120], which are: i) Requirements Elicitation, ii) Requirements Analysis & Negotiations, iii) Specifying Requirements, iv) System Modeling, v) Requirements Validation, and vi) Requirements Management [13, 22, 121];

**REPR**_**57**_ = Adopting procedures that have been discussed and agreed upon [97];

**REPR**_**58**_ = Utilizing tools which can connect with one another [107];

**REPR**_**59**_ = Utilizing the ISO/IEC TR 24766:2009 framework and related data, for evaluating the functionalities of RE tools [122, 123];

**REPR**_**60**_ = Nominating a practitioner as requirements engineer or system analyst who possesses:

- Domain knowledge or is ready to understand domain and sophisticated elicitation procedures [124].
- Capabilities for working in the global context, with remote teams and people from other cultures [124].
- Ability to settle problems and operate in unclear and uncertain conditions [124].
- Case tools, system modelling, computer languages, requirements management systems, and human-computer interface knowledge [125].
- Traits for interaction, socializing, conflict resolution, teamwork as well as individual work, creativity, and adaptability to change [126];

**REPR**_**61**_ = Selecting an appropriate requirements elicitation approach by applying a right procedure [127];

**REPR**_**62**_ = Establishing and adhering to a standardized document format [22];

**REPR**_**63**_ = For structuring the requirements description document, employing IEEE Standard 830–1998 [88];

**REPR**_**64**_ = Developing bare minimum standards to document requirements [88];

**REPR**_**65**_ = By negotiating, positioning the customer and vendor’s goals [119];

**REPR**_**66**_ = Developing a plan for RE and allocating 15 to 30 percent of overall project efforts to RE [128, 129];

**REPR**_**67**_ = Creating metrics for gauging performance [116];

**REPR**_**68**_ = Creating methods to track and report progress [116];

**REPR**_**69**_ = Raising the frequency of RE deliverables to improve progress monitoring and transparency [116];

**REPR**_**70**_ = Locating and gaining access to critical users [119, 130];

**REPR**_**71**_ = Inquiring from known or recognized partners about additional partners, creating partners’ social networks, and then ranking partners based on social network measurements [131];

**REPR**_**72**_ = Forming a Change Control Board (CCB) [102] and incorporating new requirements through an appropriate requirement change management procedure (change assessment and dissemination mechanism) [132–135];

**REPR**_**73**_ = Including actual system operators during RE process [136];

**REPR**_**74**_ = Standardizing requirements description template, by following guidelines of the IEEE Standard 830–1998 [88];

**REPR**_**75**_ = Meeting the criteria described in IEEE Standard 830–1998 in terms of quality [88];

**REPR**_**76**_ = Utilizing Wikis, physically dispersed partners are involved in the exploration of their wants, discussion of relevant issues, requests for additional characteristics, and formation of requirements [137];

**REPR**_**77**_ = Employing asynchronous communication, such as email, so that partners with lower capability have opportunity to grasp and respond to delivered messages [96, 98]. Features such as spell-checking and grammatical correction, as well as language interpretation, should really be incorporated with the email service [90];

**REPR**_**78**_ = Leveraging requirements visualization tools (such as use case and business process diagrams) and social visualization approaches to encourage partners’ participation and improve awareness about requirements [138];

**REPR**_**79**_ = Using Felder-Silverman’s Learning Style Model to choose appropriate groupware tools and strategies to elicit requirements while considering cognitive traits of partners [139];

**REPR**_**80**_ = Utilizing the same collection of tools [97];

**REPR**_**81**_ = Conducting workshops to elicit requirements [140];

**REPR**_**82**_ = Replacing conventional head-on workshops with a peer-to-peer workshop technology [141] which should offer services such as:

- Direct messaging.
- Document exchange, inspection, and modification.
- Negotiations over audio link up.
- Autonomy (by employing access privileges, a peer sends data to others while simultaneously imposing limits, such as not providing data to specific peers) provision.
- Intermittency (removal of a peer as a result of network disconnect, which can be purposeful or unintentional) detection [141];

**REPR**_**83**_ = Taking into account Hofstede’s cultural dimensions, for assisting managers in identifying individual and group conduct [109], which are:

- The distance between the power sources.
- Collectivism as opposed to individualism.
- Masculinity as opposed to Femininity.
- Avoiding uncertainty.
- Short-term opposed to Long-term adjustment [92, 142];

**REPR**_**84**_ = Encouraging informal contact amongst partners who are dispersed [143];

**REPR**_**85**_ = Making it easier for partners to communicate with one another on a regular basis [144];

**REPR**_**86**_ = Applying a proper requirements traceability method throughout the stages of requirements, design, and implementation [145];

**REPR**_**87**_ = Identifying co-change tendencies to forecast future requirements changes and developing a corresponding policy [146, 147];

**REPR**_**88**_ = Utilizing altered 100 $ method for requirements prioritization [148];

**REPR**_**89**_ = Considering that the client communication and requirements phase accounts for 10 to 25 percent of the overall project effort [149];

**REPR**_**90**_ = Forming the groups in a manner that work overlaps so that employees are aware of the other individuals’ duties [150].

The RQ1 is answered in this way.

### 4.2. Step 2: Sommerville and Sawyer’s significant RE practices to tackle issues of SDO RE process

There is a need to study which of Sommerville & Sawyer’s suggested practices are critical for addressing the RE process issues in the context of SD.

#### 4.2.1. Identification of the significant RE practices for SDO through questionnaire survey

The **S1 Appendix** presents the questionnaire employed for the survey.

**(a) Questionnaire format:** The closed format questions ask you to rank the advantages of RE techniques for SDO on a scale of one to four. The open-ended questions are designed to find out whether respondents are using any additional RE practices except the ones listed. The questionnaire is split into two sections. The first section’s goal is to gather information on the respondents’ work experience, job kind, and businesses. The second section is for data collection about key RE practices based on their assessed advantages for SDO. Two rounds of pilot study were undertaken to enhance the questionnaire layout.

The following are the various types of purported benefits of RE practices [8, 70]:

- High Perceived Benefits (H_i_): If a RE practice is mandated and always utilized, it has a "high perceived benefit".
- Medium Perceived Benefits (M_i_): If a RE practice is not obligatory but is commonly or often utilized, it has “medium perceived advantages”.
- Low Perceived Benefits (L_i_): If a RE practice is only employed for a few projects, it has "low perceived benefits".
- Zero Perceived Benefits (Z_i_): If a RE practice is never or just seldom employed, it has "zero perceived benefits".

**(b) Sampling and population:** For acquiring a valid sample of respondents, the Convenience Sampling approach was used. Project managers, software engineers, team leaders, quality assurance managers, programmers, designers, requirements engineers, analysts, and operations managers with no less than 5 years of SDO experience are among the participants. These professionals are classified as ’developers, ’managers,’ and ’senior managers,’ [71].

**(c) Response rate:** As interested professionals have been in constant communication, 45 out of 60 answers have been received by email. The 108(T) replies were chosen for data analysis, from a total of 115 (70+45) responses, grounded on the participant’s company profile, job description, and appropriate experience. Table 1 displays the results of the first questionnaire survey.

**Table 1:** Particulars of the 1^st^ questionnaire survey.

	Medium employed	No. of Questionnaires			Percentage
		Delivered	Collected back	Chosen for analysis	
	Email	60.00	45.00	**----**	75.00%
	Face-to-Face meeting	70.00	70.00	**----**	100.00%
**Overall**	**----**	130.00	115.00	**----**	88.46%
	**----**	130.00	**----**	108.00	83.08%

**(d) Criteria for choosing significant RE practices:** If at least 50% of respondents believe that a RE practice’s perceived advantages fall into the ’high perceived benefits’ and ’medium perceived benefits’ categories, then that RE practice is considered ’significant’ for resolving RE process issues for outsourced software development projects. An approach comparable to this, has been used successfully in previous investigations [70–72].

#### 4.2.2. Survey outcomes and selecting the significant RE practices

To determine the significant RE practices, out of the four classes of the RE practices’ perceived benefits, only the RE practices belonging to ’high perceived benefits’ and ’medium perceived benefits’ classes have been regarded based on the definitions of perceived benefits’ classes [8]. The Prominence Level (PL) for every RE practice indicates the percentage of replies in the ’high perceived benefits’ and ’medium perceived benefits’ classes, and is computed as specified in Eq 1:

$$PL=[(H_{i}+M_{i})/T]\times 100.$$ (1)

The survey’s findings are reported in **S2 Appendix**.

**Fig 4:**
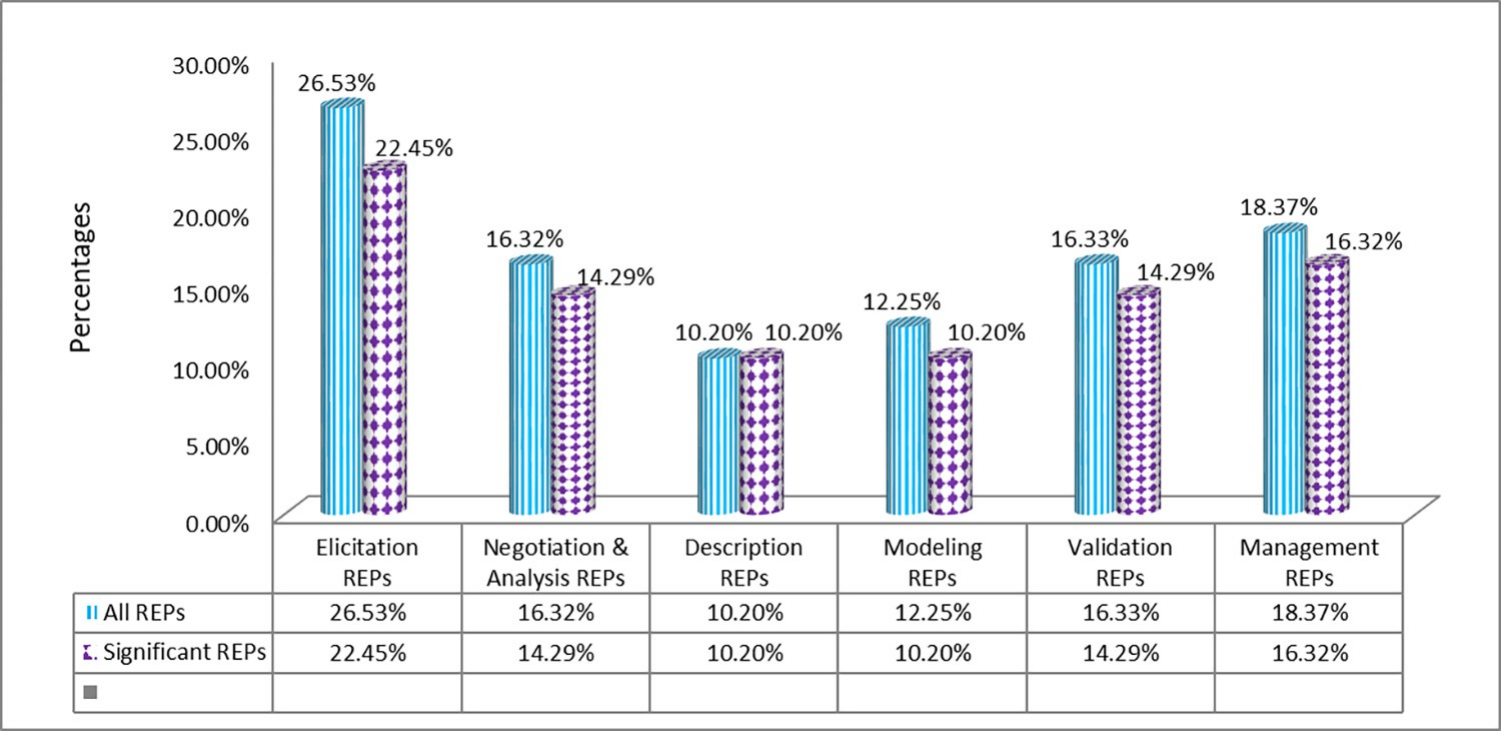
Percentages of the RE practices and significant RE Practices w.r.t phases of RE process.

The findings reveal that the bulk of the RE practices advised by Sommerville and Sawyer are significant for SDO with 43 out of 49 practices (87.76%) meeting the essential criterion. Fig 4 depicts the percentages of practices and significant practices in relation to major RE process steps.

#### 4.2.3. Significant RE practices to tackle issues of RE process for SDO

Sommerville and Sawyer’s 43 RE practices are significant for addressing the RE process issues for SDO. The 43 RE practices denoted by REPR_91_, REPR_92_, REPR_93,_ …, REPR_133_ are:

**REPR**_**91**_ = Evaluate the system’s effectiveness;

**REPR**_**92**_ = Recognizing system stakeholders and taking into account their requirements;

**REPR**_**93**_ = Sources of information must be recorded;

**REPR**_**94**_ = Determining the system’s operational environment;

**REPR**_**95**_ = Leveraging business needs to derive the requirements’ elicitation;

**REPR**_**96**_ = Search for the specific constraints of domain;

**REPR**_**97**_ = Document the justification for the requirements;

**REPR**_**98**_ = Use prototyping to understand the uncertain requirements;

**REPR**_**99**_ = Use scenarios to elicit requirements;

**REPR**_**100**_ = Outline operating procedures;

**REPR**_**101**_ = Utilize requirements from comparable systems that have already been created;

**REPR**_**102**_ = Outline boundaries in case of each system;

**REPR**_**103**_ = When assessing requirements, consider checklists;

**REPR**_**104**_ = To help negotiating, employ communication channels;

**REPR**_**105**_ = Prepare a strategy for identifying and resolving disputes;

**REPR**_**106**_ = Consultation with partners to prioritize requirements;

**REPR**_**107**_ = Categorize requirements by employing a multi-dimensional methodology;

**REPR**_**108**_ = Evaluate the risks associated with the requirements;

**REPR**_**109**_ = Develop and apply templates’ standard to describe requirements;

**REPR**_**110**_ = To express requirements adopt basic, uniform, and brief language;

**REPR**_**111**_ = Employ diagrams as needed;

**REPR**_**112**_ = Other representations for the requirements should also be incorporated to support the natural language specification;

**REPR**_**113**_ = Describe requirements numerically wherever possible;

**REPR**_**114**_ = Create a model of the system’s surroundings;

**REPR**_**115**_ = Sketch the architecture of the proposed system;

**REPR**_**116**_ = When modelling a system, utilize structured methodologies;

**REPR**_**117**_ = Prepare and utilize a data dictionary;

**PR**_**118**_ = Define the linkage between needs of the various partners and system models;

**REPR**_**119**_ = Evaluate requirements documentation to check that it adheres to your stated criteria or not;

**REPR**_**120**_ = Arranging the requirements evaluations;

**REPR**_**121**_ = Evaluating requirements by involving interdisciplinary teams;

**REPR**_**122**_ = Creating checklists to validate the requirements;

**REPR**_**123**_ = Animating the partners’ needs by utilizing prototypes;

**REPR**_**124**_ = Creating a draft version of the user manual;

**REPR**_**125**_ = System models’ rephrasing by utilizing native language;

**REPR**_**126**_ = Uniquely distinguishing every requirement of the partners;

**REPR**_**127**_ = Creating guidelines to help you manage requirements;

**REPR**_**128**_ = Establishing policies to trace various partners’ needs;

**REPR**_**129**_ = Keeping the traceability guide up to date;

**REPR**_**130**_ = Managing partners’ needs by employing database;

**REPR**_**131**_ = Creating guidelines to deal with changing needs;

**REPR**_**132**_ = Defining the system’s global needs;

**REPR**_**133**_ = Determining the needs that are prone to change.

The RQ2 is answered in this way.

### 4.3. Step 3: Identification of the additional RE practices to tackle issues of SDO RE process

Another questionnaire survey was employed for identification of the additional RE practices which are adopted by SDO professionals for addressing RE issues.

#### 4.3.1. Questionnaire survey for finding additional SDO RE practices

The **S3 Appendix** presents the questionnaire employed for the survey.

- **Questionnaire format:** The questionnaire is split into two segments. The first segment’s goal is to gather information on the participants’ work experience, job kind, and businesses. The second segment is dedicated for gathering the RE practices used by SDO practitioners to handle the issues they confront. Two rounds of pilot study were undertaken to enhance the questionnaire design.
- **Sampling and population:** For acquiring a valid sample of participants, the Convenience Sampling approach was used. Project managers, software engineers, team leaders, quality assurance managers, programmers, designers, requirements engineers, analysts, and operations managers with no less than 5 years of SDO experience are among the respondents.
- **Response rate:** There have been a total of 110 replies. The 106 replies were chosen for data analysis from 110 responses grounded on the participant’s corporate profile, job title, appropriate experience, and data reliability. Table 2 includes details about the study’s second questionnaire survey.

**Table 2:** Particulars of the 2^nd^ questionnaire survey.

		No. of Questionnaires			
	Medium employed	Delivered	Collected back	Chosen for analysis	Percentage
	Drop-Off/Pick-Up	200.00	110.00	**----**	55%
**Overall**	**----**	200.00	**----**	106.00	53%

#### 4.3.2. Consolidation of SDO RE practices

A focus group session was held with the help of two other researchers to develop the finalized listing of additional RE practices. The terminologies have been clarified, and similar RE practices from the literature and the SDO professionals have been merged.

#### 4.3.3. Additional RE practices to tackle the SDO RE process issues

The second questionnaire survey highlighted 14 additional RE practices that may be employed to solve the RE process issues for SDO. REPR_134_, REPR_135_, REPR_136,_ …, REPR_147_ denote 14 RE practices:

**REPR**_**134**_ = Motivate employees to utilize Facebook or Twitter as means of communication [Proposed];

**REPR**_**135**_ = Record synchronous communication in form of telephonic calls, Skype conversation and through videoconferencing [Proposed];

**REPR**_**136**_ = Finding and gaining access to all means of requirements which are:

- System’s end users, managers, executives, supervisors, customers, designers, and maintenance workers.
- Persons who take part in the business processes’ pursuits.
- As mentioned by client services, persons who are interested or effected.
- Client-supplied requirements or the requirements of multiple partners.
- Challenges that stakeholders encounter.
- Specialists in the field.
- Limitations, rules, and criteria that must be adhered to in the specific domain.
- Existing comparable systems.
- Consumers of the existing comparable systems.
- Records pertaining to the target system such as ledger books, bills, invoices, and notifications.
- Different applications or systems that integrate with the to-be-created system [Proposed];

**REPR**_**137**_ = Prior to actually picking RE tool(s), acquiring experience and knowledge about distinct aspects of the RE tool(s) [Proposed];

**REPR**_**138**_ = If feasible, seeking the advice of subject matter experts [Proposed];

**REPR**_**139**_ = Evaluating the likelihood of interruptions, as partners are dispersed, when estimating the time needed for various operations [Proposed];

**REPR**_**140**_ = Estimating and, if feasible, incorporating Float or Slack Time in the plan [Proposed];

**REPR**_**141**_ = In the event that development is delayed:

investing additional time and supplies;
OR after discussing with the various partners, decreasing the effort related to RE;
OR delegating some of the workload to a separate contractor [Proposed];

**REPR**_**142**_ = Establishing a list of basic criteria that will meet the customer’s demands [Proposed];

**REPR**_**143**_ = Creating a Software Requirements Specification document that everyone agrees on [Proposed];

**REPR**_**144**_ = Just share information, regarding requirements, with those who need to know [Proposed];

**REPR**_**145**_ = Assigning more funds and resources to additional needs [Proposed];

**REPR**_**146**_ = If it is impractical to adopt same operating standards or procedures, the minimal number of same operating standards or procedures should be observed [Proposed];

**REPR**_**147**_ = Notifying the customer as soon as feasible, regarding any requirement(s) that is/are not possible to accomplish [Proposed].

The RQ3 is answered in this way.

### 4.4. Exhaustive list of RE practices to tackle usually arising SDO RE process issues

To address the usually arising issues of RE process in the context of SDO, it can be seen in sections 4.1.2, 4.2.3, and 4.3.3 that:

- The present literature was used to investigate the 90 RE practices.
- Sommerville and Sawyer offer 43 RE practices, which are significant, and
- SDO industry professionals have suggested the 14 more RE practices.

As a result, there are 147(90+43+14) RE practices in the full list. Table 3 indicates the specifics.

**Table 3:** No. of identified RE practices to tackle SDO RE process issues.

RQs	Methods and sources employed	No. of RE practices
**RQ1**	Systematic literature review study was conducted to identify literature based RE practices.	90
**RQ2**	Sommerville and Sawyer’s significant RE practices were investigated using a questionnaire survey.	43
**RQ3**	To uncover further RE practices from the SDO industry, another questionnaire survey was conducted.	14
**Total**	**----**	147

This delivers a cumulative reply to RQ1, RQ2, and RQ3.

**Fig 5:**
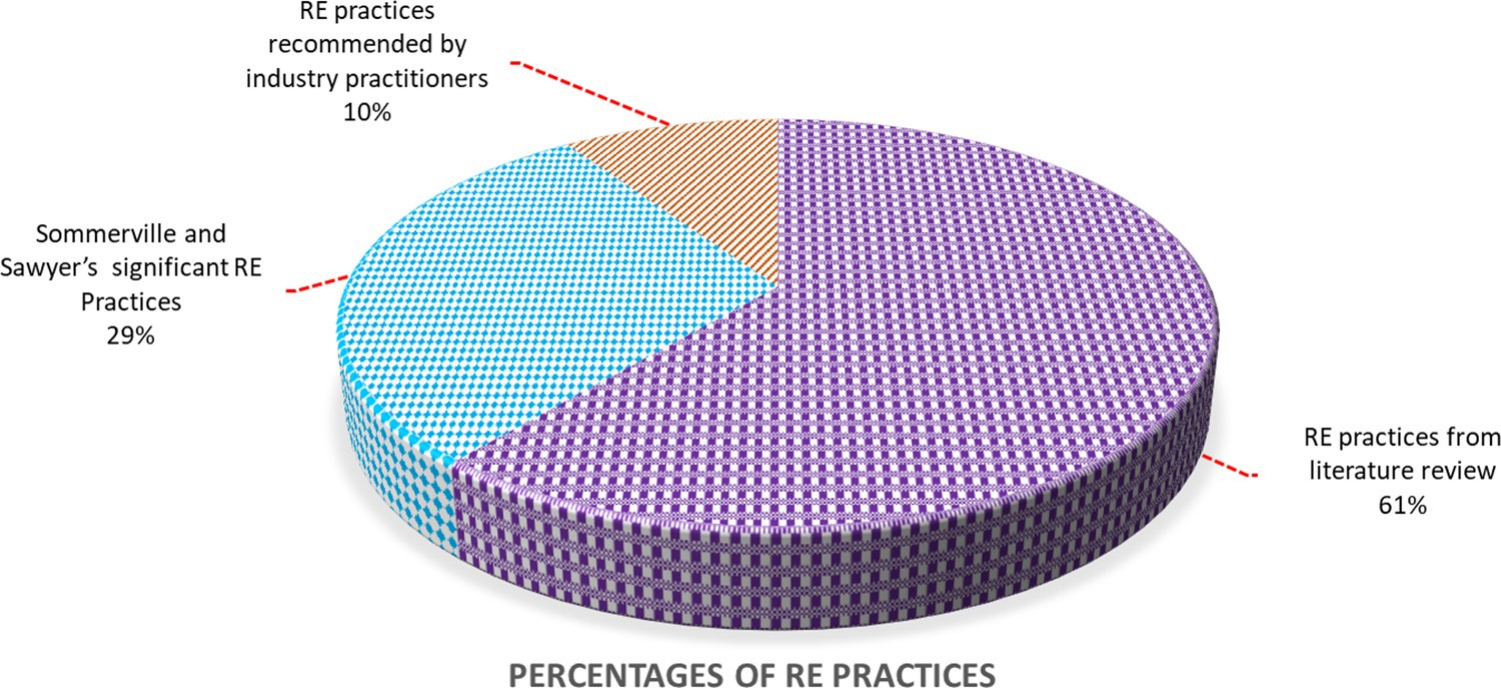
Percentages of RE practices from various resources.

The proportions of the total 147 RE practices acquired from different sources can be seen in Fig 5. As presented in Fig 5, 61 percent of RE practices were collected from the literature, 29 percent were Sommerville and Sawyer’s significant RE practices, and 10% of RE practices were proposed by SDO industrial professionals.

**S4 Appendix** comprises of an extensive compilation of 147 RE practices for tackling typical SDO RE process issues.

## 5. Ranking RE practices

To rank the RE practices, for addressing RE process issues in case of SDO, requirements prioritization techniques have been employed. Several requirements prioritization techniques exist like Analytic Hierarchy Process (AHP), Numerical Assignment, Cumulative Voting or Hundred Dollar, Bubble Sort and Ranking etc. [151–153]. To make prioritization easy and efficient, some of the requirements prioritization techniques are combined [152, 154]. For example, for Planning Game (PG), Numerical Assignment and Ranking are combined [152, 154]. For this study, Numerical Assignment and Hundred Dollar techniques have been combined. In the first step, by applying.

Numerical Assignment technique, RE practices have been divided into High, Medium, and Low groups [153, 155] based on the importance of RE practices to address SDO RE process issues. In the second step, RE practices within each group have been ranked through Hundred Dollar technique.

### 5.1. Focus group sessions

To rank the RE practices, two Focus Group sessions have been conducted and each session continued for four hours. Three experts having academic and industrial experience have participated in the sessions. The details about the participants have been provided in the Table 4.

**Table 4:** Details of focus group meeting participants.

Expert ID	Qualification	Present designation	Academic and industrial experience
Acad&prof-1	PhD	Associate Professor	11 Years
Acad&prof-2	PhD	Assistant Professor	12 Years
Acad&prof-3	MS	System Analyst	15 Years

### 5.2. Prioritizing RE practices into High, Medium, and Low groups through Numerical Assignment technique

The RE practices having High, Medium and Low importance to address SDO RE process issues, have been presented in Tables 5–7 respectively. In High importance group there are 83 RE practices, in Medium importance group there are 41 RE practices whereas in Low importance group there are 23 RE practices.

**Table 5:** RE Practices belonging to ‘High’ importance group along with respective ranks within group.

Sr. #	RE practice	Awarded dollars	Rank	Sr. #	RE practice	Awarded dollars	Rank
1	REPR_1_	90	1	43	REPR_114_	47	43
2	REPR_2_	89	2	44	REPR_136_	46	44
3	REPR_3_	88	3	45	REPR_38_	45	45
4	REPR_4_	87	4	46	REPR_39_	44	46
5	REPR_5_	86	5	47	REPR_40_	43	47
6	REPR_6_	85	6	48	REPR_41_	42	48
7	REPR_7_	84	7	49	REPR_42_	41	49
8	REPR_8_	83	8	50	REPR_43_	40	50
9	REPR_9_	82	9	51	REPR_44_	39	51
10	REPR_10_	81	10	52	REPR_48_	38	52
11	REPR_11_	80	11	53	REPR_50_	37	53
12	REPR_12_	79	12	54	REPR_53_	36	54
13	REPR_13_	78	13	55	REPR_121_	35	55
14	REPR_14_	77	14	56	REPR_122_	34	56
15	REPR_15_	76	15	57	REPR_55_	33	57
16	REPR_16_	75	16	58	REPR_56_	32	58
17	REPR_134_	73	17	59	REPR_57_	31	59
18	REPR_17_	72	18	60	REPR_59_	30	60
19	REPR_18_	71	19	61	REPR_60_	29	61
20	REPR_19_	70	20	62	REPR_138_	28	62
21	REPR_21_	69	21	63	REPR_66_	27	63
22	REPR_22_	68	22	64	REPR_139_	26	64
23	REPR_23_	67	23	65	REPR_140_	25	65
24	REPR_24_	66	24	66	REPR_85_	24	66
25	REPR_26_	65	25	67	REPR_141_	23	67
26	REPR_32_	64	26	68	REPR_70_	22	68
27	REPR_33_	63	27	69	REPR_71_	21	69
28	REPR_34_	62	28	70	REPR_72_	20	70
29	REPR_126_	61	29	71	REPR_73_	19	71
30	REPR_128_	60	30	72	REPR_98_	18	72
31	REPR_129_	59	31	73	REPR_84_	17	73
32	REPR_131_	58	32	74	REPR_101_	16	74
33	REPR_36_	57	33	75	REPR_142_	15	75
34	REPR_92_	56	34	76	REPR_103_	14	76
35	REPR_93_	55	35	77	REPR_143_	13	77
36	REPR_100_	54	36	78	REPR_99_	12	78
37	REPR_102_	53	37	79	REPR_76_	11	79
38	REPR_94_	52	38	80	REPR_78_	10	80
39	REPR_95_	51	39	81	REPR_79_	9	81
40	REPR_97_	50	40	82	REPR_81_	8	82
41	REPR_105_	49	41	83	REPR_119_	7	83
42	REPR_106_	48	42	---	---	---	---
**Total of awarded dollars**		2893				1107(1107+2893=4000)	

**Table 6:** RE Practices belonging to ‘Medium’ importance group along with respective ranks within group.

Sr. #	RE practice	Awarded dollars	Rank	Sr. #	RE practice	Awarded dollars	Rank
1	REPR_20_	45	1	22	REPR_137_	23	22
2	REPR_135_	44	2	23	REPR_61_	22	23
3	REPR_25_	43	3	24	REPR_62_	21	24
4	REPR_27_	42	4	25	REPR_63_	20	25
5	REPR_28_	41	5	26	REPR_64_	19	26
6	REPR_29_	40	6	27	REPR_65_	18	27
7	REPR_30_	39	7	28	REPR_107_	17	28
8	REPR_31_	38	8	29	REPR_67_	16	29
9	REPR_35_	37	9	30	REPR_68_	15	30
10	REPR_37_	36	10	31	REPR_69_	14	31
11	REPR_91_	35	11	32	REPR_109_	13	32
12	REPR_96_	34	12	33	REPR_110_	12	33
13	REPR_108_	33	13	34	REPR_111_	11	34
14	REPR_45_	32	14	35	REPR_112_	10	35
15	REPR_46_	31	15	36	REPR_113_	9	36
16	REPR_47_	30	16	37	REPR_123_	8	37
17	REPR_49_	28	17	38	REPR_144_	7	38
18	REPR_51_	27	18	39	REPR_145_	6	39
19	REPR_52_	26	19	40	REPR_77_	5	40
20	REPR_54_	25	20	41	REPR_122_	4	41
21	REPR_58_	24	21	---	---	---	---
**Total of awarded dollars**		730				270(270+730=1000)	

**Table 7:** RE Practices belonging to ‘Low’ importance group along with respective ranks within group.

Sr. #	RE practice	Awarded dollars	Rank	Sr. #	RE practice	Awarded dollars	Rank
1	REPR_74_	55	1	13	REPR_117_	42	13
2	REPR_75_	54	2	14	REPR_118_	41	14
3	REPR_82_	53	3	15	REPR_120_	40	15
4	REPR_83_	52	4	16	REPR_124_	39	16
5	REPR_86_	51	5	17	REPR_125_	38	17
6	REPR_87_	50	6	18	REPR_127_	37	18
7	REPR_88_	49	7	19	REPR_130_	36	19
8	REPR_89_	48	8	20	REPR_132_	35	20
9	REPR_90_	47	9	21	REPR_133_	34	21
10	REPR_104_	46	10	22	REPR_146_	33	22
11	REPR_115_	45	11	23	REPR_147_	32	23
12	REPR_116_	43	12	---	---	---	---
**Total of awarded dollars**		593				407(407+593=1000)	

### 5.3. Ranking RE practices within each group through Cumulative Voting

The ranks of the RE Practices within High, Medium, and Low groups, have been shown in Tables 5–7 respectively.

#### 5.3.1. Ranking RE practices within High importance group

For ranking of RE practices that belong to Highly important group, 4000 points or dollars have been given to each participant of the focus group sessions. After mutual discussion, the participants have unanimously awarded dollars or points to each RE practice. Based on the number of awarded dollars or points, the ranks of RE practices have been ascertained. For example, RE practice REPR_1_ has been awarded 90 dollars, REPR_2_ has been awarded 89 dollars and REPR_3_ has been awarded 88 dollars. Therefore, ranks of REPR_1,_ REPR_2_ and REPR_3_ are 1, 2 and 3 respectively as shown in the Table 5. Total of all the awarded points or dollars is 4000. Normally, 100 dollars are considered and given to each participant or stakeholder but depending upon the number of items to be ranked, more points or dollars are also deemed. For example, Regnell et al. [156] have considered 100,000 dollars to rank 75 items (58 features and 17 feature groups). Within Highly important group, ranks of all the 83 RE practices have been shown in Table 5.

#### 5.3.2. Ranking RE practices within Medium importance group

For ranking of RE practices that belong to Mediumly important group, 1000 points or dollars have been given to each participant of the focus group sessions. The participants have unanimously awarded dollars or points to each RE practice. Based on the number of awarded dollars or points, the ranks of 41 RE practices have been decided that have shown in Table 6.

#### 5.3.3. Ranking RE practices within Low importance group

For ranking of RE practices that belong to Low importance group, 1000 points or dollars have been given to each participant of the focus group sessions. The participants have unanimously awarded dollars to each RE practice. Based on the number of awarded dollars, the ranks of 23 RE practices have been decided that have shown in Table 7.

This answers RQ4.

## 6. Conclusion and future work

Because of the occurrence of Requirements Engineering (RE) process issues, the predicted benefits of software development outsourcing (SDO) are not realized in numerous instances. This study offers a collection of 147 RE practices to handle frequently occurring RE process issues. By referring 117 studies, the 90 RE practices were discovered, 43 RE practices were retrieved from the RE practices suggested by Somerville and Sawyer by contacting 108 SDO industry experts through a questionnaire survey, and 14 additional RE practices were brought out by involving 106 SDO industry practitioners through another questionnaire survey. By conducting two Focus Group sessions and applying Numerical Assignment prioritization technique, in the first step, 147 RE practices have been divided in to High, Medium, and Low importance groups. In the second step, RE practices within each group have been ranked through the Hundred Dollar technique. The suggested RE practices can be employed to tackle the commonly recurring RE process issues in the context of SDO. As a result, the study not only helps to avoid adopting haphazard RE practices for resolving frequent issues, guaranteeing RE process improvement, but also serves to actualize the predicted SDO advantages. Now next step is to develop a model for coping with the RE process issues that arise commonly in the context of SDO.

## Supporting information

S1 Appendix Questionnaire employed for the 1^st^ survey. (DOCX)

S2 Appendix Results of the 1^st^ questionnaire survey to identify Sommerville and Sawyer’s significant RE practices. (DOCX)

S3 Appendix Questionnaire employed for the 2^nd^ survey. (DOCX)

S4 Appendix Comprehensive list of 147 RE practices to address SDO RE process issues. (DOCX)
