# Supplementary material for: Towards dealing with commonly occurring requirements engineering process issues during software development outsourcing
Source: PLoS One. 2022 Jul 14;17(7):e0269607. doi: 10.1371/journal.pone.0269607 (PMC9282479; doi:10.1371/journal.pone.0269607)
Supplement: S1 Appendix — (DOCX) [file pone.0269607.s001.docx]

**Questionnaire 1: To Find Significant Requirements Engineering Practices For Software Development Outsourcing** [151]**. _________________________________________________________________________________________**

**Part 1*- Please Provide the Relevant Information / Select Appropriate Option:***

| 1. Full Name(Optional): |  | | |  |  |
| --- | --- | --- | --- | --- | --- |
| 1. Organization Name & |  | | |  |  |
| Address: |  | | |  |  |
| 1. Telephone #(Optional): |  | | |  |  |
| 1. E-mail: |  | | |  |  |
| 5 (a).**What is your position in the organization?** | | | (b).**What is your experience in outsourcing relevant jobs in the**  **current/ previous organization(s)?** | | |
|  | | |  |  |  |
|  | | |  | | |
|  | | |  | | |
|  | | |  | | |
|  | | |  | | |
| \|  \|  \| \| --- \| --- \| | | | \|  \| \| --- \| | | |
|  | | |  | | |
| 6 (a).**Number of employees in your organization?** | | (b).**What is primary business of your organization?** | |  |  |
|  | |  | |  |  |
|  | |  | |  |  |
|  | |  | |  |  |
|  | |  |  |  |  |
|  | |  | |  |  |

| 7. **What is scope of your organization?**   | |
| --- | --- |
|  | |
|  |  |

**8. What kind of software projects have you been involved?**

|  |  |
| --- | --- |
|  |  |
|  |  |
|  |  |
|  |  |
|  |  |
|  |  |
|  |  |
|  |  |
|  |  |
|  |  |

***Part 2***

| ***Outsourcing* means acquiring IT services from sources external to organization. Different IT outsourcing scenarios are:**   1. **When contractor provides services at the location of outsourcing organization.** 2. **When contractor does not provide services at the location of outsourcer but operates from the same country, it is known as *On shoring outsourcing* or *Domestic outsourcing*.** 3. **When contractor is in another country.**   **If contractor is in same region or nearby country (e.g. sharing border) then it is called *Near shoring*. But if contractor is from a far off country then it known as *Off shoring*.**   1. **When there are multiple contractors or subcontractors. If they are geographically distributed, it is called *Distributed Software Development* (DSD). When this distance becomes global, it is called *Global Software Development* (GSD).** |
| --- |

**In case of Software Development Outsourcing (SDO), vendor performs some or all of the software development activities for the client.**

**Based on your experience and perception,** **keeping in view *p*rojects that are outsourced for software development, please rank given Requirements Engineering practices based on four categories of perceived benefits:**

- ***High Perceived Benefits (H)*: A Requirement Engineering (RE) practice is referred as having ‘high perceived benefits’ if it has documented standard and is always followed as part of organization’s process to deal with outsourcing i.e., it is mandatory.**
- ***Medium Perceived Benefits (M):* A RE practice is referred as having ‘medium perceived benefits’ if it is widely followed in the organization’s process to deal with outsourcing but is not mandatory.**
- ***Low Perceived Benefits (L):* A RE practice is referred as having ‘low perceived benefits’ if it is introduced only for some projects.**
- ***Zero Perceived Benefits (Z):* A RE practice is referred as having ‘zero perceived benefits’ if it is never or rarely applied to any outsourced project.**

**2.1Requirements Documents Practices*[Use ‘X’ to select category]***

| **RE Practices** | | **H** | **M** | **L** | **Z** |
| --- | --- | --- | --- | --- | --- |
| 1. Define and follow a standard document structure. | |  |  |  |  |
| 1. Include a section in introduction part of document to explain how to use it. | |  |  |  |  |
| 1. Include a summary of the requirements. | |  |  |  |  |
| 1. Make a business case for the system. | |  |  |  |  |
| 1. Define specialized terms. | |  |  |  |  |
| 1. Lay out the document for readability. | |  |  |  |  |
| 1. Help readers to find desired information. | |  |  |  |  |
| 1. Make document easy to change. | |  |  |  |  |
| Any additional **Requirements Documents practices** other than given practices | | | | | |
|  | |  |  |  |  |
|  | |  |  |  |  |
| **2.2 Requirements Elicitation Practices*[Use ‘X’ to select category]*** | |  |  |  |  |
| **RE Practices** | | **H** | **M** | **L** | **Z** |
| 1. Assess system feasibility. | |  |  |  |  |
| 1. Sensitivity to organizational and political considerations. | |  |  |  |  |
| 1. Identifying stakeholders of system and consulting them. | |  |  |  |  |
| 1. Recording requirements originating sources. | |  |  |  |  |
| 1. Defining operating environment of system. | |  |  |  |  |
| 1. Using concerns of business for derivation of the elicitation of requirements. | |  |  |  |  |
| 1. Look for domain constraints. | |  |  |  |  |
| 1. Record requirements rationale. | |  |  |  |  |
| 1. Collect requirements from multiple viewpoints. | |  |  |  |  |
| 1. Prototype the poorly understood requirements. | |  |  |  |  |
| 1. Use scenarios to elicit requirements. | |  |  |  |  |
| 1. Define operational processes. | |  |  |  |  |
| 1. Reuse requirements from already developed similar systems. | |  |  |  |  |
| Any additional **Requirements Elicitation practices** other than given practices | | | | | |
|  | |  |  |  |  |
|  | |  |  |  |  |
|  | |  |  |  |  |
| **2.3 Requirements Analysis and Negotiation Practices*[Use ‘X’ to select category]*** | |  |  |  |  |
| **RE Practices** | | **H** | **M** | **L** | **Z** |
| 1. Define system boundaries. | |  |  |  |  |
| 1. Use checklists for requirements analysis. | |  |  |  |  |
| 1. Use communication mechanism to support negotiations. | |  |  |  |  |
| 1. Plan for conflicts identification & resolution | |  |  |  |  |
| 1. Prioritize requirements. | |  |  |  |  |
| 1. Classification of the requirements through multi-dimensional approach. | |  |  |  |  |
| 1. Using interaction matrices for finding requirements conflicts and overlaps. | |  |  |  |  |
| 1. Assess requirements risks. | |  |  |  |  |
| Any additional **Requirements Analysis and Negotiation practices** other than given practices | | | | | |
|  | |  |  |  |  |
|  | |  |  |  |  |
| **2.4Describing Requirements Practices*[Use ‘X’ to select category]*** | |  |  |  |  |
| **RE Practices** | | **H** | **M** | **L** | **Z** |
| 1. Define and use standard templates for requirements description. | |  |  |  |  |
| 1. Use simple, consistent and concise language to describe requirements. | |  |  |  |  |
| 1. Use diagrams appropriately. | |  |  |  |  |
| 1. Supplement natural language with other descriptions of the requirements. | |  |  |  |  |
| 1. Specify requirements quantitatively where appropriate. | |  |  |  |  |
| Any additional **Describing Requirements practices** other than given practices | | | | | |
|  | |  |  |  |  |
|  | |  |  |  |  |
| **2.5System Modeling Practices*[Use ‘X’ to select category]*** | |  |  |  |  |
| **RE Practices** | | **H** | **M** | **L** | **Z** |
| 1. Develop complementary system models. | |  |  |  |  |
| 1. Model the system’s environment. | |  |  |  |  |
| 1. Model the system’s architecture. | |  |  |  |  |
| 1. Use structured methods for system modeling. | |  |  |  |  |
| 1. Use a data dictionary. | |  |  |  |  |
| 1. Documentation of the association between stakeholder requirements and models of system. | |  |  |  |  |
| Any additional **System Modeling practices** other than given practices | | | | | |
|  | |  |  |  |  |
|  | |  |  |  |  |
| **2.6Requirements Validation Practices*[Use ‘X’ to select category]*** |  |  |  |  |  |
| **RE Practices** | **H** | **M** | **L** | **Z** |  |
| 1. Checking to verify that the requirements document is according to your standards. |  |  |  |  |  |
| 1. Organizing the inspections of requirements. |  |  |  |  |  |
| 1. Using multi-disciplinary teams for reviewing requirements. |  |  |  |  |  |
| 1. Defining the checklists for validation of requirements. |  |  |  |  |  |
| 1. Using prototype in order to animate the requirements. |  |  |  |  |  |
| 1. Writing a user manual draft. |  |  |  |  |  |
| 1. Proposing requirements test cases. |  |  |  |  |  |
| 1. Paraphrasing system models into natural language. |  |  |  |  |  |
| Any additional **Requirements Validation** practices other than given practices | | | | |  |
|  |  |  |  |  |  |
|  |  |  |  |  |  |
| **2.7Requirements Management Practices*[Use ‘X’ to select category]*** |  |  |  |  |  |
| **RE Practices** | **H** | **M** | **L** | **Z** |  |
| 1. Identification of each requirement uniquely. |  |  |  |  |  |
| 1. Defining policies in order to manage requirements. |  |  |  |  |  |
| 1. Defining requirements traceability policies. |  |  |  |  |  |
| 1. Maintaining the manual of traceability. |  |  |  |  |  |
| 1. Usage of database for the management of requirements. |  |  |  |  |  |
| 1. Defining policies to manage requirements change. |  |  |  |  |  |
| 1. Identification of the global system requirements. |  |  |  |  |  |
| 1. Identifying the volatile requirements. |  |  |  |  |  |
| 1. Recording of the rejected requirements. |  |  |  |  |  |
| Any additional **Requirements Management practices** other than given practices | | | | |  |
|  |  |  |  |  |  |
|  |  |  |  |  |  |
| **2.8 RE Practices for Critical Systems *[Use ‘X’ to select category]*** |  |  |  |  |  |
| **RE Practices** | **H** | **M** | **L** | **Z** |  |
| 1. Create safety requirement checklists. |  |  |  |  |  |
| 1. Involve external reviewers in the validation process. |  |  |  |  |  |
| 1. Identify and analyze hazards. |  |  |  |  |  |
| 1. Derive safety requirements from hazard analysis. |  |  |  |  |  |
| 1. Cross-check operational and functional requirements against safety requirements. |  |  |  |  |  |
| 1. Specify systems using formal specification. |  |  |  |  |  |
| 1. Record incident experiences for future use. |  |  |  |  |  |
| 1. Learn from incident experience. |  |  |  |  |  |
| 1. Establish an organizational safety culture. |  |  |  |  |  |
| Any additional **RE practices for Critical Systems** other than given practices | | | | |  |
|  |  |  |  |  |  |
|  |  |  |  |  |  |
|  |  |  |  |  |  |
|  |  |  |  |  |  |
|  |  |  |  |  |  |

***Thank you***
