# Supplementary material for: Towards dealing with commonly occurring requirements engineering process issues during software development outsourcing
Source: PLoS One. 2022 Jul 14;17(7):e0269607. doi: 10.1371/journal.pone.0269607 (PMC9282479; doi:10.1371/journal.pone.0269607)
Supplement: S2 Appendix — (DOCX) [file pone.0269607.s002.docx]

**S2 Appendix B. Results of the questionnaire survey to identify Sommerville and Sawyer’s significant RE practices for addressing issues of SDO RE process** [13,151].

1. **Results of the questionnaire survey to identify Sommerville and Sawyer’s significant RE practices**

Results of the questionnaire survey are presented based on six activities of the RE process i.e., i): Elicitation, ii): Analyzing and Negotiating, iii): Description, iv): Modeling, v): Validation, and vi): Management. Requirements Engineering Practices (REPs) have been represented by using unique Identification Numbers (IDs) from REP_1_, REP_2_, REP_3_ to REP_49_.

**1.1. Significant requirements elicitation practices**

Table 1 shows requirements elicitation practices represented by REP_n_ (n = 1, 2… 13); the frequencies of different ranks denoted by H_i_, M_i_, L_i_ and Z_i_ (i = 1, 2… 13) for high, medium, low and zero perceived benefits respectively.

There are 13 requirements elicitation practices. Practices with at least 50 Prominence Level (PL) or practices about which at least 50% of respondents think that the perceived benefits of these practices for SDO are high and medium, have been considered as significant REPs. As Table 1 shows, with the exception of REP_2_ and REP_9_, remaining 11 elicitation practices meet the prominence criterion. So REP_1_, REP_3_, REP_4_, REP_5_, REP_6_, REP_7_, REP_8_, REP_10_, REP_11_, REP_12_ and REP_13_ are significant requirements elicitation practices for SDO.

**Table 1. Significant requirements elicitation practices**

| **IDs** | **Practices** | **Assessed ranks** | | | | **PL** |
| --- | --- | --- | --- | --- | --- | --- |
|  |  | **H_i_** | **M_i_** | **L_i_** | **Z_i_** |  |
| REP_1_ | Assess system feasibility. | 29 | 37 | 20 | 22 | 61.11 |
| REP_2_ | Sensitivity to organizational and political considerations. | 21 | 28 | 29 | 30 | 45.37 |
| REP_3_ | Identifying stakeholders of system and consulting them. | 27 | 40 | 24 | 17 | 62.04 |
| REP_4_ | Recording requirements originating sources. | 32 | 36 | 21 | 19 | 62.96 |
| REP_5_ | Defining operating environment of system. | 31 | 38 | 21 | 18 | 63.89 |
| REP_6_ | Using concerns of business for derivation of the elicitation of requirements. | 36 | 34 | 20 | 18 | 64.81 |
| REP_7_ | Look for domain constraints. | 42 | 29 | 25 | 12 | 65.74 |
| REP_8_ | Record requirements rationale. | 43 | 29 | 27 | 09 | 66.67 |
| REP_9_ | Collect requirements from multiple viewpoints. | 34 | 16 | 35 | 23 | 46.30 |
| REP_10_ | Prototype the poorly understood requirements. | 40 | 35 | 19 | 14 | 69.44 |
| REP_11_ | Use scenarios to elicit requirements. | 50 | 23 | 24 | 11 | 67.59 |
| REP_12_ | Define operational processes. | 42 | 29 | 25 | 12 | 65.74 |
| REP_13_ | Reuse requirements from already developed similar systems. | 46 | 23 | 24 | 15 | 63.89 |

- 1. **Significant requirements analysis and negotiation practices**

Table 2 shows 8 requirements analysis and negotiation practices represented by REP_n_ (n = 14, 15,…, 21); the frequencies of different ranks denoted by H_i_, M_i_, L_i_ and Z_i_ (i = 14, 15,…, 21) for high, medium, low and zero perceived benefits respectively.

As shown in Table 2, out of 8 requirements analysis and negotiation practices, 7 practices are significant as they have PLs 50 or above which means that according to the opinion of 50% or more respondents these practices have high and medium benefits for SDO. So REP_14_, REP_15_, REP_16_, REP_17_, REP_18_, REP_19_ and REP_21_ are significant requirements analysis and negotiation practices for SDO.

**Table 2. Significant requirements analysis and negotiation practices**

| **IDs** | **Practices** | **Assessed ranks** | | | | **PL** |
| --- | --- | --- | --- | --- | --- | --- |
|  |  | **H_i_** | **M_i_** | **L_i_** | **Z_i_** |  |
| REP_14_ | Define system boundaries. | 31 | 35 | 19 | 23 | 61.11 |
| REP_15_ | Use checklists for requirements analysis. | 29 | 36 | 20 | 23 | 60.19 |
| REP_16_ | Use communication mechanism to support negotiations. | 34 | 37 | 20 | 17 | 65.74 |
| REP_17_ | Plan for conflicts identification & resolution. | 33 | 36 | 18 | 21 | 63.89 |
| REP_18_ | Prioritize requirements. | 50 | 51 | 07 | 0 | 93.52 |
| REP_19_ | Classification of the requirements through multi-dimensional approach. | 32 | 36 | 18 | 22 | 62.96 |
| REP_20_ | Using interaction matrices for finding requirements conflicts and overlaps. | 25 | 19 | 40 | 24 | 40.74 |
| REP_21_ | Assess requirements risks. | 34 | 25 | 27 | 22 | 54.63 |

- 1. **Significant requirements’ description practices**

Table 3 shows 5 requirements description practices represented by REP_n_ (n = 22, 23,..., 26); the frequencies of different ranks denoted by H_i_, M_i_, L_i_ and Z_i_ (i = 22, 23,…, 26) for high, medium, low and zero perceived benefits respectively.

Table 3 provides data about the requirements description practices. All the 5 requirements description practices i.e., REP_22_, REP_23_, REP_24_, REP_25_ and REP_26_ are significant or important for SDO as for all these practices PLs are 50 or above indicating that 50% or more respondents perceive them as having high and medium benefits for SDO.

**Table 3. Significant requirements description practices**

| **IDs** | **Practices** | **Assessed ranks** | | | | **PL** |
| --- | --- | --- | --- | --- | --- | --- |
|  |  | **H_i_** | **M_i_** | **L_i_** | **Z_i_** |  |
| REP_22_ | Define and use standard templates for requirements description. | 34 | 35 | 19 | 20 | 63.89 |
| REP_23_ | Use simple, consistent and concise language to describe requirements. | 32 | 36 | 17 | 23 | 62.96 |
| REP_24_ | Use diagrams appropriately. | 36 | 35 | 19 | 18 | 65.74 |
| REP_25_ | Supplement natural language with other descriptions of the requirements. | 39 | 33 | 19 | 17 | 66.67 |
| REP_26_ | Specify requirements quantitatively where appropriate. | 31 | 38 | 20 | 19 | 63.89 |

- 1. **Significant system modeling practices**

Table 4 shows 6 system modeling practices represented by REP_n_ (n = 27, 28,…, 32); the frequencies of different ranks denoted by H_i_, M_i_, L_i_ and Z_i_  (i = 27, 28,…, 32) for high, medium, low and zero perceived benefits respectively.

As shown in Table 4, there are 6 system modeling practices i.e., REP_27_, REP_28,_ REP_29_, REP_30_, REP_31_ and REP_32_. Out of these 6 modeling practices, only one practice which is REP_27_ is regarded as unimportant for SDO as the PL for the REP_27_ is 48.15 showing that 50% or more respondents do not consider this practice as having high and medium benefits for SDO.

The remaining 5 practices i.e., REP_28_, REP_29_, REP_30_, REP_31_ and REP_32_ possess PLs above 50. Therefore, they are significant or substantial system modeling practices for SDO.

**Table 4. Significant requirements modeling practices**

| **IDs** | **Practices** | **Assessed ranks** | | | | **PL** |
| --- | --- | --- | --- | --- | --- | --- |
|  |  | **H_i_** | **M_i_** | **L_i_** | **Z_i_** |  |
| REP_27_ | Develop complementary system models. | 22 | 30 | 36 | 20 | 48.15 |
| REP_28_ | Model the system’s environment. | 38 | 35 | 19 | 16 | 67.59 |
| REP_29_ | Model the system’s architecture. | 37 | 39 | 20 | 12 | 70.37 |
| REP_30_ | Use structured methods for system modeling. | 40 | 44 | 13 | 11 | 77.78 |
| REP_31_ | Use a data dictionary. | 36 | 38 | 19 | 15 | 68.52 |
| REP_32_ | Documentation of the association between stakeholder requirements and models of system. | 40 | 40 | 20 | 08 | 74.07 |

- 1. **Significant requirements validation practices**

Table 5 shows 8 requirements validation practices represented by REP_n_ (n = 33, 34… 40); the frequencies of different ranks denoted by H_i_, M_i_, L_i_ and Z_i_ (i = 33, 34… 40) for high, medium, low and zero perceived benefits respectively.

This can be observed from Table 5 that out of 8 requirements validation practices, 7 practices i.e., REP_33_, REP_34_, REP_35_, REP_36_, REP_37_, REP_38_ and REP_40_ have the least required PLs. Only one validation practice that is REP_39_ is regarded as trivial for SDO because the PL for this practice is 47.22, which is insufficient to meet the required criterion. So REP_33_, REP_34_, REP_35_, REP_36_, REP_37_, REP_38_ and REP_40_ are significant or important requirements validation practices for SDO.

**Table 5. Significant requirements validation practices**

| **IDs** | **Practices** | **Assessed ranks** | | | | **PL** |
| --- | --- | --- | --- | --- | --- | --- |
|  |  | **H_i_** | **M_i_** | **L_i_** | **Z_i_** |  |
| REP_33_ | Checking to verify that the requirements document is according to your standards. | 40 | 36 | 20 | 12 | 70.37 |
| REP_34_ | Organizing the inspections of requirements. | 39 | 36 | 19 | 14 | 69.44 |
| REP_35_ | Using multi-disciplinary teams for reviewing requirements. | 42 | 35 | 19 | 12 | 71.30 |
| REP_36_ | Defining the checklists for validation of requirements. | 30 | 40 | 28 | 10 | 64.81 |
| REP_37_ | Using prototype in order to animate the requirements. | 38 | 36 | 21 | 13 | 68.52 |
| REP_38_ | Writing a user manual draft. | 41 | 37 | 21 | 09 | 72.22 |
| REP_39_ | Proposing requirements test cases. | 26 | 25 | 38 | 19 | 47.22 |
| REP_40_ | Paraphrasing system models into natural language. | 41 | 35 | 17 | 15 | 70.37 |

- 1. **Significant requirements management practices**

Table 6 shows 9 requirements management practices represented by REP_n_ (n = 41, 42… 49); the frequencies of different ranks denoted by H_i_, M_i_, L_i_ and Z_i_ (i = 41, 42… 49) for high, medium, low and zero perceived benefits respectively.

This can be observed from Table 6 that for requirements management there are 9 practices_._ Only one management practice that is REP_49_ has PL less than 50 (46.30) which is not enough to become a significant RE practice for SDO. All the other 8 practices to manage requirements have PLs above 50 indicating that more than 50% of respondents consider these practices as having high and medium benefits for SDO. Therefore, REP_41_, REP_42_, REP_43_, REP_44_, REP_45_, REP_46_, REP_47_ and REP_48_ are significant requirements management practices for SDO.

**Table 6. Significant requirements management practices**

| **IDs** | **Practices** | **Assessed ranks** | | | | **PL** |
| --- | --- | --- | --- | --- | --- | --- |
|  |  | **H_i_** | **M_i_** | **L_i_** | **Z_i_** |  |
| REP_41_ | Identification of each requirement uniquely. | 30 | 36 | 19 | 23 | 61.11 |
| REP_42_ | Defining policies in order to manage requirements. | 34 | 35 | 24 | 15 | 63.89 |
| REP_43_ | Defining requirements traceability policies. | 40 | 37 | 21 | 10 | 71.30 |
| REP_44_ | Maintaining the manual of traceability. | 44 | 32 | 20 | 12 | 70.37 |
| REP_45_ | Usage of database for the management of requirements. | 33 | 36 | 30 | 09 | 63.89 |
| REP_46_ | Defining policies to manage requirements change. | 42 | 36 | 20 | 10 | 72.22 |
| REP_47_ | Identification of the global system requirements. | 40 | 30 | 25 | 13 | 64.81 |
| REP_48_ | Identifying the volatile requirements. | 36 | 32 | 23 | 17 | 62.96 |
| REP_49_ | Recording of the rejected requirements. | 14 | 36 | 28 | 30 | 46.30 |
