# Supplementary material for: Towards dealing with commonly occurring requirements engineering process issues during software development outsourcing
Source: PLoS One. 2022 Jul 14;17(7):e0269607. doi: 10.1371/journal.pone.0269607 (PMC9282479; doi:10.1371/journal.pone.0269607)
Supplement: S3 Appendix — (DOCX) [file pone.0269607.s003.docx]

**Questionnaire 2:**  **To find issues, faced by Software Development Outsourcing Industry, of Requirements Engineering process for Software Development Outsourcing and RE Practices to address those issues** [151].

**________________________________________________________________________________**

**Part 1*- Please Provide the Relevant Information / Select Appropriate Option***

| 1. Full Name(Optional): |  | | |  |  |
| --- | --- | --- | --- | --- | --- |
| 1. Organization Name & |  | | |  |  |
| Address: |  | | |  |  |
| 1. Telephone #(Optional): |  | | |  |  |
| 1. E-mail: |  | | |  |  |
| 5 (a).**What is your position in the organization?** | | | (b).**What is your experience in outsourcing relevant jobs in the**  **current/ previous organization(s)?** | | |
|  | | |  |  |  |
|  | | |  | | |
|  | | |  | | |
|  | | |  | | |
|  | | |  | | |
| \|  \|  \| \| --- \| --- \| | | | | | |
|  | | |  | | |
| 6 (a).**Number of employees in your organization?** | | (b).**What is primary business of your organization?** | | |  |
|  | |  | | |  |
|  | |  | | |  |
|  | |  | | |  |
|  | |  |  | |  |
|  | |  | | |  |

| 7. **What is scope of your organization?**   | |
| --- | --- |
|  | |
|  |  |

**8. What kind of software projects have you been involved?**

|  |  |
| --- | --- |
|  |  |
|  |  |
|  |  |
|  |  |
|  |  |
|  |  |
|  |  |
|  |  |
|  |  |
|  |  |

***Part 2***

**We want to purpose a model to address the issues of Requirements Engineering (RE) process for Software Development Outsourcing (SDO). During the career, you would have been facing issues that affect RE process for outsourced software development projects. Those issues are of various types and thus belong to different categories. For example, you may have issues while communicating with other stakeholders, you may face issues because of improper knowledge management or you can come across cultural issues as different stakeholders belong to different backgrounds, speak different languages and live in different parts of world. A literature-based list of such issues has been provided with respect to various categories. The different categories of issues are 1: Communication, 2: Knowledge** [**management and awareness**](http://www.um.es/giisw/GSD/wiki/index.php/Knowledge_management_and_awareness)**, 3:** [**Cultural diversities**](http://www.um.es/giisw/GSD/wiki/index.php/Cultural_differences)**, 4:** [**Management and coordination**](http://www.um.es/giisw/GSD/wiki/index.php/Management_and_project_coordination)**, 5: Processes and tools, 6: Relationship among stakeholders, and 7: Requirements centric.**

**A):** Based on your perception and experience if you feel that an issue should be placed in a category other than the present one then please mention that issue, new category and reason for category change in the space provided at the end of each category**.**

**B):** If you have ever faced issue(s) other than given in case of each category OR you perceive that there are the issues that may arise during the RE process for SDO but have not been provided in the category-wise literature-based list of the issues then please mention**: i).** Those issues **ii).** Relevant RE practices to address corresponding issues, in the space provided at the end of each category**.**

| **Category #1: Communication Issues** | | |
| --- | --- | --- |
| 1. Infrequent and constrained communication among the stakeholders during RE process. | | |
| 1. Lack of informal communication among the stakeholders during RE process. | | |
| 1. For clarification and resolution of misunderstandings about requirements, face to face communication is required. | | |
| 1. Lack of face to face communication. | | |
| 1. Lack of synchronous communication. | | |
| 1. Even through the videoconferencing, it is hard to conduct long and productive negotiations, in particular when many stakeholders are involved. | | |
| 1. Delayed responses. | | |
| 1. Arrangement of collocated meetings among stakeholders is unfeasible in most of cases because of the distance involved. | | |
| 1. Poor client-vendor communication. | | |
| 1. Arranging the face-to-face gatherings escalates cost. | | |
| 1. Stakeholders do not use synchronous Internet communication technologies to communicate about the requirements and depend on formal means like scheduled meetings, emails and documents. | | |
| 1. Unproductive meetings that are held to take decisions about the requirements. | | |
| 1. Asynchronous communication causes delay in propagation and solution of issues. | | |
| 1. When there is synchronous meeting among the sites having considerable time differences, the stakeholders at least from one site are quite disturbed since either it is too late or too early with respect to daily working schedule. | | |
| 1. Stakeholders are not fluent in the communication language. | | |
| 1. Electronic communication like email allows covert communication that creates challenges for resolving requirements conflicts. | | |
| 1. Stakeholders do not communicate with each other effectively and look for exerting power and using influence on each other. | | |
| 1. For clarification and resolution of issues, any team member can communicate with any other stakeholder that can result in repetitive discussions and demands for extra controlling efforts. | | |
| 1. Communication gaps or delays during RE due to personality clashes. | | |
| 1. Online communication for clarification of requirements leads to problematic requirements as they are ambiguous, change frequently or are incomplete. | | |
| 1. Attaining the consent of distant stakeholders for interviewing and scheduling it. | | |
| **To Change the Category of Issues, if Needed** | | |
| **Issue** | **New Category** | **Reason for Changing Category** |
|  |  |  |
|  |  |  |
|  |  |  |
| **Any Issue For Communication Category Other Than Given Issues** | | |
| **Sr. No** | **Issue** | **Practices to address Issues** |
|  |  |  |
|  |  |  |
|  |  |  |
| **Category #2 : Knowledge** [**management and awareness**](http://www.um.es/giisw/GSD/wiki/index.php/Knowledge_management_and_awareness) **Issues** | | |
| 1. Hindrance in circulation of requirements knowledge from/to organizations. | | |
| 1. Incapability of tracing the stakeholders, and related information, that are affected by introducing new requirements. | | |
| 1. Stakeholders cannot find the relevant knowledge, integration of the procedures, which can be used for knowledge synthesis, is inappropriate and knowledge transfer is blocked or delayed. | | |
| 1. Unawareness of the stakeholders from current/latest information about requirements. | | |
| 1. 5. Requirements information obtained by multiple distant sources is not shared with all the stakeholders. | | |
| 1. Geographically distributed stakeholders cannot get the advantages of social mechanisms and processes that are present in case of co-located workspace, therefore, need for awareness about the requirements is intensified. | | |
| 1. Reopening of the already discussed and seemingly settled issues. | | |
| 1. Organizational structure, that does not match with the task assignments, can hinder the flow of knowledge. | | |
| 1. Propagation of the information about requirements changes is ineffective. | | |
| 1. Practitioners forget to inform relevant stakeholders about the requirements changes. | | |
| 1. The groups that are working on the same or associated requirements are not aware of the stakeholders affected by alterations in the requirements or stakeholders who affect the requirement changes. | | |
| 1. Poor requirements change management. | | |
| 1. The various teams/groups that are working on the identical or associated requirements are not aware of the expertise of practitioners from remote groups. | | |
| 1. Formal means of communication like documents cannot exhibit requirements’ changes as rapidly as it may be essential. | | |
| 1. Working on obsolete requirements. | | |
| 1. Availability of the consistent information, as sources are distributed, is difficult. | | |
| 1. Lack of the awareness, about the environment in which system is to be deployed, may lead to ambiguous requirements. | | |
| 1. Unawareness from the context and importance of requirements leading to project delays and quality compromises. | | |
| 1. Requirements clarifications are conveyed late that cause delay in project. | | |
| 1. Inability of sharing knowledge or best practices. | | |
| 1. Unawareness of requirements engineers from the effects of new system implementation on the client organization. | | |
| **To Change the Category of Issues, if Needed** | | |
| **Issue** | **New Category** | **Reason for Changing Category** |
|  |  |  |
|  |  |  |
|  |  |  |
| **Any Issue For Knowledge** [**management and awareness**](http://www.um.es/giisw/GSD/wiki/index.php/Knowledge_management_and_awareness) **Category Other Than Given Issues** | | |
| **Sr. No** | **Issue** | **Practices to address Issues** |
|  |  |  |
|  |  |  |
|  |  |  |
| **Category # 3:** [**Cultural Diversities’**](http://www.um.es/giisw/GSD/wiki/index.php/Cultural_differences) **Issues** | | |
| 1. Distance causes cultural differences among the various functional units of an organization that creates hindrance in attaining the common understanding of requirements. | | |
| 1. Creating trust is challenging. | | |
| 1. Maintaining trust is challenging. | | |
| 1. Lack of trust. | | |
| 1. Avoidance of the commitments from the stakeholders. | | |
| 1. Loss of cohesion among stakeholders because of geographical dispersion. | | |
| 1. Difficulties in achieving consensus on requirements. | | |
| 1. Stakeholders belonging to diverse cultural backgrounds have different ethical values about hierarchies, handling risks, following schedules and punctuality that may escalate conflicts. | | |
| 1. Different cultures have different ethics regarding precision of work and improvisation ability. | | |
| 1. Practitioners from diverse cultural backgrounds have inexplicit and unstated meanings and explanations of the information about the requirements. | | |
| 1. Practitioners from various cultural backgrounds draw/deduce varied meanings from messages. | | |
| 1. Some practitioners, because of their cultural backgrounds, cannot disagree with the clients, therefore, ‘nice to have’ requirements and key requirements have equal priorities. | | |
| 1. Customer requirements are not well-understood and delivered because of dissimilar culture and language. | | |
| 1. Participants of remote requirements engineering meetings are not expert in single common language. | | |
| 1. Stakeholders are at different proficiency level of communication language, therefore, stakeholder at higher level dominates and affects the requirements communication. | | |
| 1. Same words are used to convey the different meanings in different organizations that creates misunderstandings for specifying and validating requirements. | | |
| 1. The individuals, who are not proficient in communication language, are reluctant in asking questions for clarifications. | | |
| 1. Shyness of the stakeholders, for example avoidance from making phone calls to unfamiliar persons, causes delayed communication. | | |
| 1. The requirements comprehension is reduced if requirements are stated in the non-native language. | | |
|  | | |
|  | | |
|  | | |
|  | | |
|  | | |
|  | | |
| **To Change the Category of Issues, if Needed** | | |
| **Issue** | **New Category** | **Reason for Changing Category** |
|  |  |  |
|  |  |  |
|  |  |  |
| **Any Issue For**  **Cultural Diversities Category Other Than Given Issues** | | |
| **Sr. No** | **Issue** | **Practices to address Issues** |
|  |  |  |
|  |  |  |
|  |  |  |
| **Category #4:** [**Management and coordination**](http://www.um.es/giisw/GSD/wiki/index.php/Management_and_project_coordination) **Issues** | | |
| 1. Difficulties in comprehending information, reasons and activities that are required for common Requirements Understanding (RU) among the dispersed stakeholders. | | |
| 1. Time zone differences cause problems for coordination. | | |
| 1. Hindrance for appropriate involvement of stakeholders in RE activities because of time differences. | | |
| 1. Delay in clarifications about requirements and decision making. | | |
| 1. Propensity of less or non-reporting of the problems because of distance. | | |
| 1. Even the skilled professionals can become nervous and inactive because of distance. | | |
| 1. Poorly defined or undefined responsibilities. | | |
| 1. Absence of central and trusted management in area of requirements engineering causing poor coordination. | | |
| 1. Absence of a firm, skilled and central analyst role. | | |
| 1. Underestimation of the time required for conducting requirements review. | | |
| 1. Unfair allocation of workload to various teams. | | |
| 1. No assessment of the effect of distribution on different RE activities. | | |
| 1. Conflicting interests of different stakeholders. | | |
| 1. The requirements are elicited from the stakeholders that are spread over various organizational units and thus requirements have to bundle or pack. | | |
| 1. High number of stakeholders as sources of requirements. | | |
| 1. Need for adjustment of actual requirements to interact with other software(s). | | |
| 1. Analysts change requirements by ignoring the change management process. | | |
| 1. In case of temporal dispersion, synchronized coordination is required to build the trust. | | |
| 1. Remote teams or RE teams work with tight schedules to meet deadlines. | | |
| **To Change the Category of Issues, if Needed** | | |
| **Issue** | **New Category** | **Reason for Changing Category** |
|  |  |  |
|  |  |  |
|  |  |  |
| **Any Issue For** [**Management and coordination**](http://www.um.es/giisw/GSD/wiki/index.php/Management_and_project_coordination) **Category Other Than Given Issues** | | |
| **Sr. No** | **Issue** | **Practices to address Issues** |
|  |  |  |
|  |  |  |
|  |  |  |
| **Category #5: Processes and tools’ Issues** | | |
| 1. Lack of clearly defined RE process. | | |
| 1. The stakeholders employ dissimilar processes for analyzing and documenting requirements. | | |
| 1. Stakeholders employ different processes to manage changes in requirements. | | |
| 1. The documented processes may not be applied. | | |
| 1. Use of different RE processes, resulting in different templates and methodologies, at the different locations of client. | | |
| 1. Use of unsuitable RE processes. | | |
| 1. Some team members do not attend the requirements engineering meetings as they are not familiar with the tools and technologies being used. | | |
| 1. The tools cannot be integrated with other tools. | | |
| 1. RE rework or data loss during transfer from one tool to other. | | |
| 1. Need of the tools that provide permanent access to the requirements related information. | | |
| 1. Tools do not convey requirements change information to the relevant stakeholders at the appropriate time. | | |
| 1. Need of the tools that facilitate traceability of requirements across borders of tools. | | |
| 1. Need of the tools that support requirements negotiations among the remote stakeholders. | | |
| 1. Tools lack the facility of requirements document evolution through the collaboration among remote stakeholders. | | |
| 1. Selection of inappropriate RE tool(s). | | |
| 1. Lack of training for using groupware tools. | | |
| **To Change the Category of Issues, if Needed** | | |
| **Issue** | **New Category** | **Reason for Changing Category** |
|  |  |  |
|  |  |  |
|  |  |  |
| **Any Issue For Processes and Tools Category Other Than Given Issues** | | |
| **Sr. No** | **Issue** | **Practices to address Issues** |
|  |  |  |
|  |  |  |
|  |  |  |
| **Category #6: Relationship among stakeholders Issues** | | |
| 1. Lack of firm relationship among stakeholders. | | |
| 1. Information about the identifications or resolutions of requirements’ issues is not conveyed to other sites for a long time. | | |
| 1. Intermittency of informal contacts causes less opportunity of building relationships. | | |
| 1. Use of different requirements documentation standards by customer and vendor. | | |
| 1. Formation of customer and/or vendor teams on ad hoc basis. | | |
| 1. Different priorities of client and vendor for collecting and finalizing requirements. | | |
| 1. Lack of participation, in RE process, from client side. | | |
| 1. Misconceptions of the vendor teams about client’s working practices. | | |
| 1. Client and vendor follow conflicting requirements engineering approaches. | | |
| 1. Failure in meeting deadlines and fulfilling commitments about requirements by vendor. | | |
| 1. Issues in signing-off requirements engineering deliverables. | | |
| 1. Differences on selection of requirements engineering tools. | | |
| 1. Customers think that performing requirements related work from remote locations is not possible. | | |
| 1. Client and vendor rely on oral agreement. | | |
| **To Change the Category of Issues, if Needed** | | |
| **Issue** | **New Category** | **Reason for Changing Category** |
|  |  |  |
|  |  |  |
|  |  |  |
| **Any Issue For Relationship Among Stakeholders Category Other Than Given Issues** | | |
| **Sr. No** | **Issue** | **Practices to address Issues** |
|  |  |  |
|  |  |  |
|  |  |  |
|  |  |  |
|  |  |  |
|  |  |  |
|  |  |  |
| **Category #7: Requirements centric Issues** | | |
| 1. Finalize requirements for all stakeholders based on the requirements gathered or information obtained from the available stakeholders. | | |
| 1. Requirements (specifications) are misinterpreted. | | |
| 1. Incorrect or false requirements. | | |
| 1. Requirements are not based upon appropriate/ sound business case. | | |
| 1. Gold plating or extra requirements. | | |
| 1. Incomplete requirements. | | |
| 1. Requirements documentation without following any standard. | | |
| 1. Requirements are out of the scope of project. | | |
| 1. Poor or ambiguous requirements specification. | | |
| 1. Not providing information or providing intentionally ambiguous information about requirements. | | |
| 1. How prioritization of requirements should be done. | | |
| 1. Requirements change frequently. | | |
| 1. Requirements document becomes inconsistent. | | |
| 1. Requirements are stretched resulting in scope creeping. | | |
| 1. Requirements elicitation through fragmentation, that is requirements related to different parts of system are elicited by different people who work independently, leads to customer dissatisfaction. | | |
| 1. Analysts are familiar with the domestic projects but do not possess the skills required for dealing with requirements specification in case of outsourcing. | | |
| 1. Only selected stakeholders are consulted during the requirements elicitation that results in biased elicitation. | | |
| 1. System users and people who interact with the requirements engineering team are different. | | |
| 1. Pressure on Requirements Engineers to hide certain information about requirements, resulting in compromised requirements elicitation and specification. | | |
| **To Change the Category of Issues, if Needed** | | |
| **Issue** | **New Category** | **Reason for Changing Category** |
|  |  |  |
|  |  |  |
|  |  |  |
| **Any Issue For Requirements Category Other Than Given Issues** | | |
| **Sr. No** | **Issue** | **Practices to address Issues** |
|  |  |  |
|  |  |  |
|  |  |  |

***Thank You***
