# Supplementary material for: Towards dealing with commonly occurring requirements engineering process issues during software development outsourcing
Source: PLoS One. 2022 Jul 14;17(7):e0269607. doi: 10.1371/journal.pone.0269607 (PMC9282479; doi:10.1371/journal.pone.0269607)
Supplement: S4 Appendix — (DOCX) [file pone.0269607.s004.docx]

**S4 Appendix D.** **Comprehensive list of 147 RE practices to address SDO RE process issues**

{Containing literature-based practices (REPR_1_-REPR_90_), Sommerville and Sawyer’s significant practices (REPR_91_-REPR_133_) and additional practices (REPR_134_-REPR_147_)} [151].

| **Identified practices** | **Literature-based practices to address the issues of RE process issues for SDO** |
| --- | --- |
| **REPR_1_** | Putting in place the necessary infrastructure to support communication and guaranteeing that it is operational [87]; |
| **REPR_2_** | Promoting synchronous communication using chat rooms, phone conversations, and videoconferencing [87]; |
| **REPR_3_** | Adjusting to and comprehending various stakeholders' cultures [87], means being familiar with a culture's customs, values, ethos, and local language [5]; |
| **REPR_4_** | Choosing and utilizing a standardized communication language [88]; |
| **REPR_5_** | Concentrating on communication language improvement, such as English language classes [87, 89, 90]**;** |
| **REPR_6_** | Employing cultural liaisons [87, 89, 91] or intermediaries (people who are conversant with customer and vendor cultures) [92]**;** |
| **REPR_7_** | Creating a 'proximity training center' in a time zone that is the same or somewhat different from the client's zone [93]; |
| **REPR_8_** | Attempting to identify natural overlaps in work time [94]; |
| **REPR_9_** | Evaluating a person's capacity to work 'around the clock' [94]; |
| **REPR_10_** | **REPR_10_=** Time-shifting (adjusting one's working time to coincide with the work time of others) to attain time zone closeness, a variety of approaches for this purpose are:   1. Flextime (functioning on a flexible schedule to allow for overlapping). 2. Overtime (functioning for additional time for overlapping). 3. Telework (functioning for elastic schedules from home for overlapping). 4. Long working days (allowing for work time overlapping at the beginning or finish of the day).   v) Unrestricted working hours (employees decide their own operating time for overlapping and there are no  standard work hours) [95]**;** |
| **REPR_11_** | Providing electronic message "drop in", remote phoning, and artefact distribution capabilities to distant practitioners' rooms [96]; |
| **REPR_12_** | Enabling professional integration from the outset of the project, such as by holding face-to-face kick-off meetings to create personal interactions [21, 97]; |
| **REPR_13_** | Organizing regular visits to isolated locations in order to foster trust [18, 98, 99]; |
| **REPR_14_** | Encouraging direct contact amongst stakeholders [100]; |
| **REPR_15_** | Assuring that stakeholders are introduced to each other from the start of the project [101]; |
| **REPR_16_** | Facilitating interaction in the client's original tongue [5]; |
| **REPR_17_** | Advising on the usage of groupware tools [99]; |
| **REPR_18_** | Attempting to convince stakeholders that disclosing concerns or sharing information would have beneficial repercussions rather than adverse effects [94]; |
| **REPR_19_** | Organizing video or teleconferences [117] on day-to-day, weekly, semi-monthly, or monthly basis such that no or a few awkward hours exist for all the partners [98]; |
| **REPR_20_** | Organizing requirements engineering sessions by:   1. Using a human moderator and a rich communication medium that allows data, audios, and videos to be integrated. 2. Making and sticking to an agenda. 3. Identifying appropriate participants and notifying them on time to participate in requirements engineering sessions. 4. Exchanging of reference materials in a timely manner to allow participants ample time to view the essential content. 5. Giving attendees of requirements sessions access to resources (like emails, relevant papers, work artefacts, and so on) containing information on the requirements [21]; |
| **REPR_21_** | Setting up assertive governance at the project manager and team leader levels [102]; |
| **REPR_22_** | Keeping track of the directives in a certain order [102]; |
| **REPR_23_** | Personal and group obligations must be clearly stated and agreed upon [102]**;** |
| **REPR_24_** | Getting obviously defined and grasped requirements engineering processes [102]; |
| **REPR_25_** | Utilizing email as a communication verification tool because it preserves a written copy of contact [21, 89, 96]; |
| **REPR_26_** | Getting agreements in writing and adequately documented [103]; |
| **REPR_27_** | Creating an organizational structure with clear communication roles [91]; |
| **REPR_28_** | At the various levels of team, project, and management; creating peer-to-peer connectivity across remote locations [91]; |
| **REPR_29_** | Partly orchestrating inter-organizational procedures [91]; |
| **REPR_30_** | Providing open channels of communication amongst stakeholders with clearly defined responsibilities [91]; |
| **REPR_31_** | Assessing and communicating development on jointly approved artefacts on a regular basis [91]; |
| **REPR_32_** | Making use of an awareness support system to manage requirements, all partners ought to be able to obtain the following details:   1. Specifications, justifications, and priorities of various requirements. 2. Requirements dependencies, as well as design, code, and testing dependencies. 3. Duties of every member of the team in relation to specific requirement(s) and contact particulars like email address and phone number. 4. The people who took initiatives for the requirements. 5. Issues relating to requirements, issues’ activators, state of issues’ resolution, and judgments made because of issues. 6. Dates, times, and venues of the meetings, as well as the present stakeholders, debated issues and judgments done. 7. Demands for change, change demand activators, state of every change demand, personnel engaged in making judgments, and judgments made [104]; |
| **REPR_33_** | Retaining senior professionals on the team and persuading those professionals to bridge the knowledge gap [105]**;** |
| **REPR_34_** | Putting in place a centralized communication system [105]; |
| **REPR_35_** | After each meeting, making a report of the proceedings. Any member of the team or moderator ought to outline which issues were presented at the meeting, what decisions were taken about every issue, still open issues, who is responsible for gathering more data, and whose guidance should be solicited in the event of each issue [106]; |
| **REPR_36_** | To use a Requirements Management System (to regulate and follow changes) that has the succeeding characteristics:   1. Searching a list of requirements, extracting individual requirements, and organizing requirements according to certain criteria. 2. Administration of the requirements modification process, assistance for requirements traceability, and development of various forms of requirements reporting. 3. Acceptance of external documents via an interface. 4. Managing several versions of requirements. 5. Aid for carrying out various forms of analyses (e.g., impact analysis, knowing whether a requirement is orphan, status tracking). 6. Limiting access and editing privileges to the list of requirements [29]**;** |
| **REPR_37_** | Notifying the pertinent partner regarding the change in requirements by:   1. Communication technologies such as telephone, emails, and the internet. 2. Using the system to send out automated notifications [107]**;** |
| **REPR_38_** | In the event that there are a large number of partners:   1. Designating a person (communication channel) out of each organizational unit or group of requirements sources to collect requirements from that unit or group. The requirements are then transferred to an expert via communication channels, where they might be combined [108]. 2. Obtaining unanimity on requirements employing group elicitation approaches like Brainstorming, JAD, Focus groups, and requirements Workshops [25]. 3. Generating a consolidated requirements document that includes all of the requirements [108]**;** |
| **REPR_39_** | Adopting the following steps to address cultural challenges:   1. (REPR_6_) Employing cultural liaisons [87, 89, 91] or intermediaries (people who are conversant with customer and vendor cultures) [92]. 2. Advising members of the team to tour other partners' sites [109]. 3. Organizing cultural training sessions [109]. 4. Offering cross-cultural orientation sessions [109]. 5. Considering stakeholders' cultural beliefs when determining female responsibilities [109]. 6. Introducing a 'Negotiated Culture,' a negotiated culture created to respect all partners' cultural standards [103]. 7. Identifying persons with expertise and familiarity with the customer's culture to support for requirements discussion and definition [89]. 8. (REPR_4_) Choosing and utilizing a standardized communication language [88]. 9. (REPR_5_) Concentrating on communication language improvement, such as English language classes [87, 89, 90]. 10. The project manager or/and experienced team members planning and supervision of all the efforts that are carried out to cope with cultural differences [109]; |
| **REPR_40_** | Presenting the Equality Model (EM) for all partners, in which all partners are treated equally and can discuss each other's preferences, religion, and social traditions. They can also exchange knowledge and make recommendations based on others' perspectives and roles [110]; |
| **REPR_41_** | Defining the procedures, techniques, and policies that must be followed [111]**;** |
| **REPR_42_** | Knowledge exchange [111]**;** |
| **REPR_43_** | Observance of common hopes [111]**;** |
| **REPR_44_** | Possessing technological, managerial, and personnel capabilities for satisfying quality standards & meeting deadlines [5]; |
| **REPR_45_** | To inspire non-fluent or less confident partners to participate in the discourse, begin with an informal dialogue [112]; |
| **REPR_46_** | Making use of translation facilities:   1. Utilizing human interpreter [112, 113]. 2. Utilizing real-time machine conversion facilities [113]**;** |
| **REPR_47_** | Utilizing scales to calculate the mean time it takes for hopes to be met. For example, providing a feature that determines the average time it takes a person or team to react to an email. If the mean time to respond is three days, the sender may anticipate receiving a response within three days [114]; |
| **REPR_48_** | Developing and utilizing a vocabulary and notations for requirements description [88]; |
| **REPR_49_** | Vendor managers can take the following steps to establish collaboration:   1. Establishing team members' roles and tasks, as well as constructing Organizational Charts that show their roles and obligations [115]. 2. Obtaining and administering the appropriate human resources using Resource Calendar [115]. 3. Appropriate task distribution [115]. 4. (REPR_28_) At the various levels of team, project, and management; creating peer-to-peer connectivity across remote locations [91]. 5. (REPR_29_) Partly orchestrating inter-organizational procedures [91]. 6. (REPR_30_) Providing open channels of communication amongst stakeholders with clearly defined responsibilities [91]. 7. (REPR_31_) Assessing and communicating development on jointly approved artefacts on a regular basis [91]; |
| **REPR_50_** | Achieving partners’ agreement on meeting attendance terms and conditions, as well as fulfilling timelines and obligations  [116]**;** |
| **REPR_51_** | Identifying each team member's function and suggesting who might interact with whom [16, 117]; |
| **REPR_52_** | In terms of decisions, keep in constant contact with customers by organizing:   1. Meetings in person. 2. Video conferencing [16]**;** |
| **REPR_53_** | Selecting a teammate that works beyond the typical business hours and responds to questions [118]**;** |
| **REPR_54_** | Educating on in what manner to:   1. Make use of the available tools. 2. Interact efficiently in a situation where partners are scattered at remote sites [18]**;** |
| **REPR_55_** | Giving prospective team members training about how to use relevant procedures, as well as associated tools and technology [119]; |
| **REPR_56_** | Performing six general steps for RE, in absence of any standard RE process [20, 120], which are :i) Requirements Elicitation, ii) Requirements Analysis & Negotiations, iii) Specifying Requirements, iv) System Modeling, v) Requirements Validation, and vi) Requirements Management [13, 22, 121]**;** |
| **REPR_57_** | Adopting procedures that have been discussed and agreed upon [97]**;** |
| **REPR_58_** | Utilizing tools which can connect with one another [107]**;** |
| **REPR_59_** | Utilizing the ISO/IEC TR 24766:2009 framework & related data, for evaluating the functionalities of RE tools [122, 123]**;** |
| **REPR_60_** | Nominating a practitioner as requirements engineer or system analyst who possesses:   1. Domain knowledge or is ready to understand domain and sophisticated elicitation procedures [124]. 2. Capabilities for working in the global context, with remote teams and people from other cultures [124]. 3. Ability to settle problems and operate in unclear and uncertain conditions [124]. 4. Case tools, system modelling and computer languages, requirements management systems, and human-computer interface knowledge [125]. 5. Traits for interaction, socializing, conflict resolution, teamwork as well as individual work, creativity, and adaptability to change [126]**;** |
| **REPR_61_** | Selecting an appropriate requirements elicitation approach by applying a right procedure [127]**;** |
| **REPR_62_** | Establishing and adhering to a standardized document format [22]**;** |
| **REPR_63_** | For structuring the requirements description document, employing IEEE Standard 830-1998 [88]**;** |
| **REPR_64_** | Developing bare minimum standards to document requirements [88]**;** |
| **REPR_65_** | By negotiating, positioning the customer and vendor's goals [119]; |
| **REPR_66_** | Developing a plan for RE and allocating 15 to 30 percent of overall project efforts to RE [128,129]**;** |
| **REPR_67_** | Creating metrics for gauging performance [116]**;** |
| **REPR_68_** | Creating methods to track and report progress [116]**;** |
| **REPR_69_** | Raising the frequency of RE deliverables to improve progress monitoring and transparency [116]**;** |
| **REPR_70_** | Locating and gaining access to critical users [119, 130]**;** |
| **REPR_71_** | Inquiring from known or recognized partners about additional partners, creating partners' social networks, and then ranking partners based on social network measurements [131]; |
| **REPR_72_** | Forming a Change Control Board (CCB) [102] and incorporating new requirements through an appropriate requirement change management procedure (change assessment and dissemination mechanism) [132-135]; |
| **REPR_73_** | Including actual system operators during RE process [136]**;** |
| **REPR_74_** | Standardizing requirements description template, by following guidelines of the IEEE Standard 830-1998 [88]**;** |
| **REPR_75_** | Meeting the criteria described in IEEE Standard 830-1998 in terms of quality [88]**;** |
| **REPR_76_** | Utilizing Wikis, physically dispersed partners are involved in the exploration of their wants, discussion of relevant issues, requests for additional characteristics, and formation of requirements [137]; |
| **REPR_77_** | Employing asynchronous communication, such as email, so that partners with lower capability have opportunity to grasp and respond to delivered messages [96, 98]. Features such as spell-checking and grammatical correction, as well as language interpretation, should really be incorporated with the email service [90]; |
| **REPR_78_** | Leveraging requirements visualization tools (such as use case and business process diagrams) and social visualization approaches to encourage partners’ participation and improve awareness about requirements [138]; |
| **REPR_79_** | Using Felder-Silverman’s Learning Style Model to choose appropriate groupware tools and strategies to elicit requirements while considering cognitive traits of partners [139]; |
| **REPR_80_** | Utilizing the same collection of tools [97]**;** |
| **REPR_81_** | Conducting workshops to elicit requirements [140]**;** |
| **REPR_82_** | Replacing conventional head-on workshops with a peer-to-peer workshop technology [141] which should offer services such as:   1. Direct messaging. 2. Document exchange, inspection, and modification. 3. Negotiations over audio link up. 4. Autonomy (by employing access privileges, a peer sends data to others while simultaneously imposing limits, such as not providing data to specific peers) provision. 5. Intermittency (the removal of a peer as a result of network disconnect, which can be purposeful or unintentional) detection [141]**;** |
| **REPR_83_** | Taking into account Hofstede's cultural dimensions, for assisting managers in identifying individual and group conduct [109], which are:   1. The distance between the power sources. 2. Collectivism as opposed to individualism. 3. Masculinity as opposed to Femininity. 4. Avoiding uncertainty. 5. Short-term opposed to Long-term adjustment [92, 142]; |
| **REPR_84_** | Encouraging informal contact amongst partners who are dispersed [143]; |
| **REPR_85_** | Making it easier for partners to communicate with one another on a regular basis [144]**;** |
| **REPR_86_** | Applying a proper requirements traceability method throughout the stages of requirements, design, and implementation [145]**;** |
| **REPR_87_** | Identifying co-change tendencies to forecast future requirements changes and developing a corresponding policy [146, 147]**;** |
| **REPR_88_** | Utilizing altered 100 $ method for requirements prioritization [148]**;** |
| **REPR_89_** | Considering that the client communication and requirements phase accounts for 10 to 25 percent of the overall project effort [149]**;** |
| **REPR_90_** | Forming the groups in a manner that work overlaps so that employees are aware of the other individuals' duties [150]**.** |

| **Identified practices** | **Sommerville and Sawyer’ significant RE practices for SDO** |
| --- | --- |
| **REPR_91_** | Evaluate the system's effectiveness |
| **REPR_92_** | Recognizing system stakeholders and taking into account their requirements**;** |
| **REPR_93_** | Sources of information must be recorded**;**. |
| **REPR_94_** | Determining the system's operational environment**;** |
| **REPR_95_** | Leveraging business needs to derive the requirements’ elicitation**;** |
| **REPR_96_** | Search for the specific constraints of domain**;** |
| **REPR_97_** | Document the justification for the requirements; |
| **REPR_98_** | Use prototyping to understand the uncertain requirements**;** |
| **REPR_99_** | Use scenarios to elicit requirements**;** |
| **REPR_100_** | Outline operating procedures**;** |
| **REPR_101_** | Utilize requirements from comparable systems that have already been created**;** |
| **REPR_102_** | Outline boundaries in case of each system**;** |
| **REPR_103_** | When assessing requirements, consider checklists**;** |
| **REPR_104_** | To help negotiating, employ communication channels**;** |
| **REPR_105_** | Prepare a strategy for identifying and resolving disputes**;** |
| **REPR_106_** | Consultation with partners to prioritize requirements**;** |
| **REPR_107_** | Categorize requirements by employing a multi-dimensional methodology**;** |
| **REPR_108_** | Evaluate the risks associated with the requirements**;** |
| **REPR_109_** | Develop and apply templates’ standard to describe requirements; |
| **REPR_110_** | To express requirements adopt basic, uniform, and brief language**;** |
| **REPR_111_** | Employ diagrams as needed**;** |
| **REPR_112_** | Other representations for the requirements should also be incorporated to support the natural language specification; |
| **REPR_113_** | Describe requirements numerically wherever possible**;** |
| **REPR_114_** | Create a model of the system's surroundings**;** |
| **REPR_115_** | Sketch the architecture of the proposed system**;** |
| **REPR_116_** | When modelling a system, utilize structured methodologies**;** |
| **REPR_117_** | Prepare and utilize a data dictionary**;** |
| **REPR_118_** | Define the linkage between needs of the various partners and system models**;** |
| **REPR_119_** | Evaluate requirements documentation to check that it adheres to your stated criteria or not**;** |
| **REPR_120_** | Arranging the requirements evaluations**;** |
| **REPR_121_** | Evaluating requirements by involving interdisciplinary teams**;** |
| **REPR_122_** | Creating checklists to validate the requirements; |
| **REPR_123_** | Animating the partners’ needs by utilizing prototypes**;** |
| **REPR_124_** | Creating a draft version of the user manual**;** |
| **REPR_125_** | System models’ rephrasing by utilizing native language**;** |
| **REPR_126_** | Uniquely distinguishing every requirement of the partners**;** |
| **REPR_127_** | Creating guidelines to help you manage requirements**;** |
| **REPR_128_** | Establishing policies to trace various partners’ needs**;** |
| **REPR_129_** | Keeping the traceability guide up to date**;** |
| **REPR_130_** | Managing partners’ needs by employing database**;** |
| **REPR_131_** | Creating guidelines to deal with changing needs**;** |
| **REPR_132_** | Defining the system's global needs**;** |
| **REPR_133_** | Determining the needs that are prone to change; |
| **Identified practices** | **Additional practices, reported by SDO Practitioners, to address the issues of RE process for SDO** |
| **REPR_134_** | Motivate employees to utilize Facebook or Twitter as means of communication [Proposed]**;** |
| **REPR_135_** | Record synchronous communication in form of telephonic calls, Skype conversation and through videoconferencing [Proposed]**;** |
| **REPR_136_** | Finding and gaining access to all means of requirements which are:  i) System’s end users, managers, executives, supervisors, customers, designers, and maintenance workers.  ii) Persons who take part in the business processes’ pursuits.  iii) As mentioned by client services, persons who are interested or effected.  iv) Client-supplied requirements or the requirements of multiple partners.  v) Challenges that stakeholders encounter.  vi) Specialists in the field.  vii) Limitations, rules, and criteria that must be adhered to in the specific domain.  viii) Existing comparable systems.  ix) Consumers of the existing comparable systems.  x) Records pertaining to the target system such as ledger books, bills, invoices, and notifications.  xi) Different applications or systems that integrate with the to-be-created system [Proposed]**;** |
| **REPR_137_** | Prior to actually picking RE tool(s), acquiring experience and knowledge about distinct aspects of the RE tool(s) [Proposed]**;** |
| **REPR_138_** | If feasible, seeking the advice of subject matter experts [Proposed]**;** |
| **REPR_139_** | Evaluating the likelihood of interruptions, as partners are dispersed, when estimating the time needed for various operations [Proposed]**;** |
| **REPR_140_** | Estimating and, if feasible, incorporating Float or Slack Time in the plan [Proposed]**;** |
| **REPR_141_** | In the event that development is delayed:  investing additional time and supplies;  OR after discussing with the various partners, decreasing the effort related to RE;  OR delegating some of the workload to a separate contractor [Proposed]**;** |
| **REPR_142_** | Establishing a list of basic criteria that will meet the customer's demands [Proposed]**;** |
| **REPR_143_** | Creating a Software Requirements Specification document that everyone agrees on [Proposed]**;** |
| **REPR_144_** | Just share information, regarding requirements, with those who need to know [Proposed]**;** |
| **REPR_145_** | Assigning more funds and resources to additional needs [Proposed]**;** |
| **REPR_146_** | If it is impractical to adopt same operating standards or procedures, the minimal number of same operating standards or procedures should be observed [Proposed]**;** |
| **REPR_147_** | Notifying the customer as soon as feasible, regarding any requirement(s) that is/are not possible to accomplish [Proposed]**.** |
